# Golgi stress mediates redox imbalance and ferroptosis in human cells

**DOI:** 10.1038/s42003-018-0212-6

**Published:** 2018-11-28

**Authors:** Hamed Alborzinia, Tatiana I. Ignashkova, Francesca R. Dejure, Mathieu Gendarme, Jannick Theobald, Stefan Wölfl, Ralph K. Lindemann, Jan H. Reiling

**Affiliations:** 1 BioMed X Innovation Center, Im Neuenheimer Feld 583, 69120 Heidelberg, Germany; 2 Institute of Pharmacy and Molecular Biotechnology, Heidelberg University, Im Neuenheimer Feld 364, 69120 Heidelberg, Germany; 3 Translational Innovation Platform Oncology, Merck Biopharma, Merck KGaA, Frankfurter Str. 250, 64293 Darmstadt, Germany; 4 Present Address: Division of Stem Cells and Cancer, German Cancer Research Center (DKFZ) and Heidelberg Institute for Stem Cell Technology and Experimental Medicine (HI-STEM), Im Neuenheimer Feld 280, 69120 Heidelberg, Germany; 5 Present Address: Institute for Applied Cancer Science and Center for Co-Clinical Trials, University of Texas MD Anderson Cancer Center, Houston, TX USA

**Keywords:** Small molecules, Cell death, Cell signalling

## Abstract

Cytotoxic activities of several Golgi-dispersing compounds including AMF-26/M-COPA, brefeldin A and golgicide A have previously been shown to induce autophagy or apoptosis. Here, we demonstrate that these Golgi disruptors also trigger ferroptosis, a non-apoptotic form of cell death characterized by iron-dependent oxidative degradation of lipids. Inhibitors of ferroptosis not only counteract cell death, but they also protect from Golgi dispersal and inhibition of protein secretion in response to several Golgi stress agents. Furthermore, the application of sublethal doses of ferroptosis-inducers such as erastin and sorafenib, low cystine growth conditions, or genetic knockdown of SLC7A11 and GPX4 all similarly protect cells from Golgi stress and lead to modulation of ACSL4, SLC7A5, SLC7A11 or GPX4 levels. Collectively, this study suggests a previously unrecognized function of the Golgi apparatus, which involves cellular redox control and prevents ferroptotic cell death.

## Abstract (web summary)

Hamed Alborzinia et al. show that Golgi-dispersing compounds trigger iron-dependent oxidative degradation of lipids, inducing a non-apoptotic cell death called ferroptosis. This study provides insight into the role of Golgi apparatus for preventing ferroptotic cell death through its cellular redox control.

## Introduction

Regulated execution of cell death and elimination of harmful cells during development and in response to insurmountable cell stress is critical for multicellular organisms to prevent the onset of malignancy^1^. Arguably, apoptotic cell death is currently best understood among other known forms of programmed cell death. The use of small molecules has been instrumental to elucidate additional cell death programs^2–4^. However, a better understanding of pharmacologically induced cell death processes will be crucial for the design of more specific drugs, to overcome secondary resistance in response to small molecule treatment and to devise more effective drug combination strategies. Cell death is likely to be governed in a context and cell type-specific manner^5^ and in many instances will be the result of interconnected cell death cascades^4^. Recently, a new form of regulated necrotic cell death-termed ferroptosis was described^6^. This non-apoptotic mechanism of cell death requires iron and is morphologically, genetically and biochemically distinct from other cell death pathways^7^. Ferroptotic cell death is characterized by iron-dependent lipid peroxidation ultimately leading to oxidative cell death by overwhelming the cellular antioxidant defense. Several pharmacological ferroptosis inducers have been described. One class, which includes erastin, glutamate, and FDA-approved drugs such as sorafenib, sulfasalazine, and artemisinin derivatives, causes inhibition of the plasma membrane antiporter known as system x_c_^−^, which transports extracellular cystine (Cys_2_, which can be intracellularly reduced to cysteine) into the cell in exchange for intracellular glutamate. Inhibition of cystine import leads to depletion of total glutathione (GSH), which is critical for the protection against oxidative stress. In mammals, the nonessential amino acid cysteine can either be obtained through uptake or, alternatively, through de novo synthesis via the transsulfuration pathway, which generates cysteine through the conversion of methionine as sulfur donor via the intermediates homocysteine and cystathionine^8–10^. Relatively little is known about the importance and regulation of the transsulfuration pathway for the generation of cysteine/glutathione in normal and stressed cells, but it has the capacity to act as backup system under oxidative stress conditions^10,11^. Another class of ferroptosis-inducing compounds such as RAS-selective lethal 3 (RSL3) acts more downstream by directly inhibiting the catalytic activity of the selenoprotein glutathione peroxidase 4 (GPX4) that normally reduces lipid and organic hydroperoxides to their respective alcohols and thereby defends against membrane lipid peroxidation and oxidative damage. Due to the requirement of GSH as cosubstrate of GPX4 in the reduction process of phospholipid hydroperoxides^12^, both classes of ferroptosis inducers ultimately block GPX4 activity resulting in excessive generation of oxidized lipids. Another key factor involved in ferroptosis execution is acyl-CoA synthetase long-chain family member 4 (ACSL4), which catalyzes esterification of fatty acyls such as arachidonoyl and adrenoyl into phosphatidylethanolamines before the phosphatidylethanolamine species are either enzymatically or nonenzymatically converted into ferroptotic death signals by lipoxygenase family members or by free-radical chain reactions involving Fenton chemistry, respectively. Importantly, ACSL4 expression levels are predictive of cellular ferroptosis sensitivity^13,14^.

Multiple reports have demonstrated the ability of brefeldin A (BFA) to induce apoptosis in various cancer cell lines independently of their *p53* status^15–19^. Similar to BFA, golgicide A (GCA), and AMF-26 (also called M-COPA) are Golgi disruptors and reversible inhibitors of ARF1-GBF1 with a mode of action comparable to BFA^20–23^. However, a refined picture of the cell death programs triggered downstream of these Golgi stress-inducing compounds has not been elucidated. In addition, it is unknown whether BFA can activate alternative cell death mechanisms besides apoptosis and autophagy^24^.

Here, we find that in multiple human cell lines Golgi-dispersing agents including BFA, GCA, AMF-26 or AG1478/tyrphostin induce ferroptosis. Accumulation of lipid peroxides, a reduction in the intracellular glutathione pool and changes in expression levels of several ferroptosis signaling components are observed following Golgi stress. Furthermore, antioxidants, iron chelators, and reactive oxygen species (ROS) scavengers as well as overexpression of glutathione S-transferase alpha 1 (GSTA1), SLC7A11 and GPX4, or ACSL4 knockdown protect cells from Golgi stress-mediated cell death. Notably, BFA-induced Golgi dispersal, suppression of protein secretion, endoplasmic reticulum (ER) stress or DNA damage is prevented by ferroptosis inhibitor co-treatment suggesting that the control of lipid ROS formation is critical for secretory pathway homeostasis. On the other hand, overexpression of the Golgi-associated small GTPase ADP ribosylation factor 1 (ARF1) is sufficient to counteract BFA-induced lipid peroxide formation. Unexpectedly, similar to ferroptosis inhibitors, several ferroptosis inducers such as erastin or sorafenib, used at nontoxic concentrations unable to elicit discernable lipid peroxidation in cells, prevent Golgi stress-induced dispersal and lethality, which is dependent on the transsulfuration pathway. Further, shRNA-mediated knockdown of SLC7A11 or GPX4 results in enhanced viability upon BFA treatment, which might be caused by concomitant ACSL4 downregulation and by reduced autophagy levels in these cells.

## Results

### Golgi stress-inducing compounds cause ROS formation

**Fig. 1:**
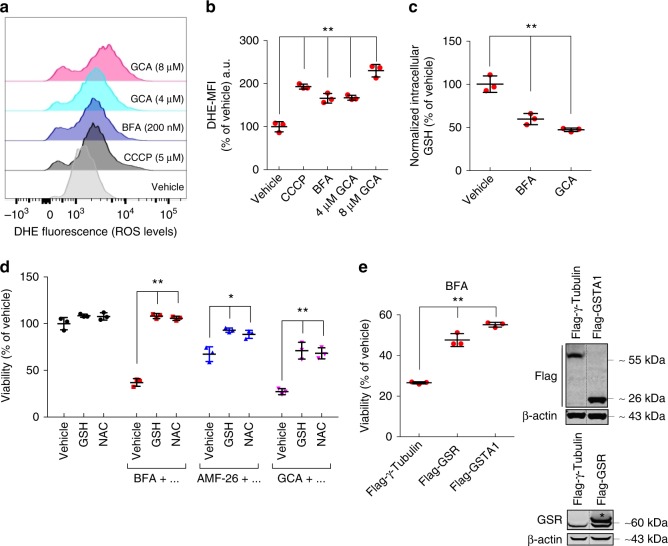
ROS induction and depletion of glutathione in response to Golgi stress. **a** Analysis of intracellular reactive oxygen species (ROS) levels using dihydroethidium (DHE) staining and flow cytometry of HeLa cells treated for 48 h with the indicated compounds. CCCP was used as a positive control. **b** Quantification of the relative DHE mean fluorescence intensity (MFI) levels of samples shown in the FACS histogram in **a**; **P < 0.01; a.u. arbitrary unit. **c** Quantification of total intracellular glutathione (GSH) levels in HeLa cells treated with 60 nM BFA or 2 µM GCA for 48 h; **P < 0.01. **d** Relative viability (survival of compound-treated cells divided by survival of vehicle-treated cells) of HeLa cells after treatment with BFA (35 nM), AMF-26 (35 nM) or GCA (1.5 µM) for 72 h in the presence or absence of glutathione (GSH, 2 mM) or *N*-acetyl cysteine (NAC, 1 mM) as measured by the CellTiter-Blue (CTB) assay; *P < 0.05 and **P < 0.01. **a**–**d**) Shown are representative examples of at least three independent experiments, and three wells per treatment condition were measured. Center bars indicate the mean, error bars indicate the SD. **e** Displayed is the relative mean cell viability ±SD of HeLa cells stably overexpressing Flag-γ-Tubulin, Flag-GSR or Flag-GSTA1 following treatment with 35 nM BFA for 72 h; **P < 0.01. Cell survival was measured by the CTB assay. Shown is a representative example of three independent experiments, and three wells per genotype and treatment condition were measured. Expression levels of overexpressed proteins are shown by immunoblotting. Protein lysates were run on the same gel, and dashed lines in blots indicate where irrelevant samples were cropped out. **a**–**e** Statistical analysis was performed using Student’s two-tailed *t*-test. Scanned images of unprocessed blots are shown in Supplementary Fig. 7

**Fig. 2:**
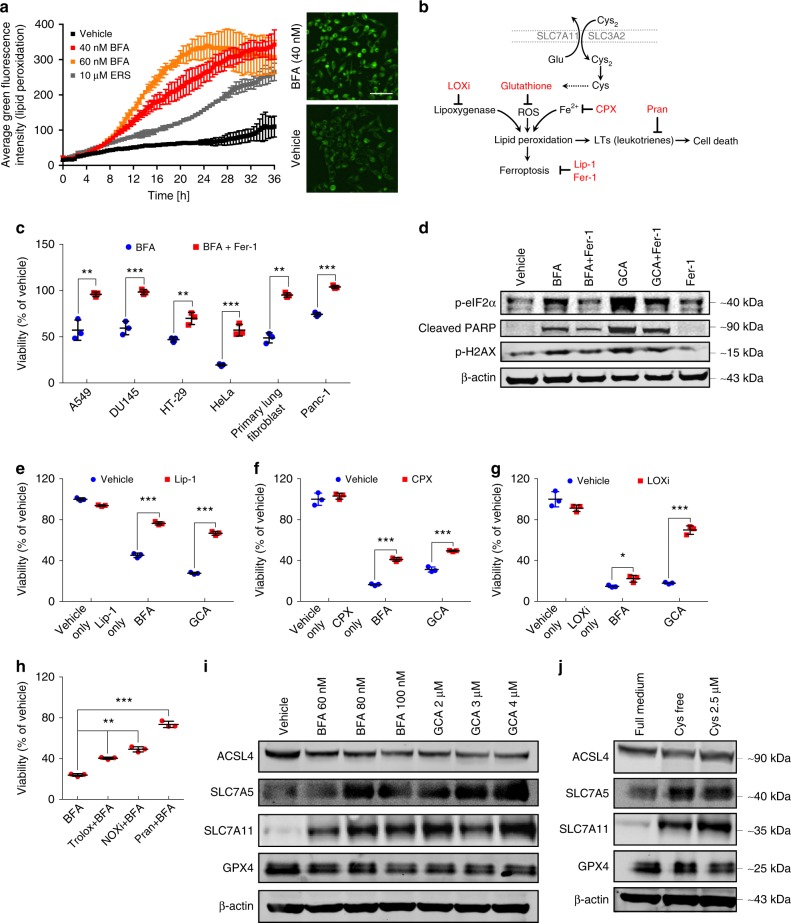
Golgi stress leads to ferroptosis. **a** Real-time lipid peroxidation analysis using the IncuCyte system and Liperfluo staining of HeLa cells treated with vehicle or the indicated concentrations of BFA and erastin (ERS), respectively. Immunofluorescence images of HeLa cells treated for 20 h with 40 nM BFA and stained with Liperfluo are shown on the right; scale bar: 50 µm. Shown is a representative example of three independent experiments. Bars and error bars represent the means and SD, respectively. Statistical significance between the different treatment conditions was calculated using a two-way ANOVA test, and the average fluorescence intensity for each condition was derived from four pictures taken per well from four separate wells. BFA treatment (both concentrations) caused significant differences in lipid peroxide production compared to vehicle treatment starting at ten hours of treatment; *P* < 0.01. **b** Schematic illustration of Cys_2_ uptake/metabolism and GSH biosynthesis and the role of iron and GSH in ferroptosis induction. A number of known ferroptosis/ROS inhibitors are shown (CPX, Fer-1, Lip-1, LOXi, Pran). **c** Relative viability of a panel of cancer cell lines as well as primary lung fibroblasts after treatment with BFA for 72 h in the presence or absence of 10 µM ferrostatin-1 (Fer-1); **P < 0.01; ***P < 0.001. BFA concentrations used: HeLa: 35 nM; A549, DU145, HT-29 and primary lung fibroblast: 60 nM; Panc-1: 120 nM. **d** Western blot analysis of HeLa cells that were either vehicle-treated, treated with 35 nM BFA or with 1.5 µM GCA in the presence or absence of 10 µM ferrostatin-1 (Fer-1) for 24 h. β-actin was used as loading control. **e**, **f** Relative viability of HeLa cells after treatment with 35 nM BFA or 1.5 µM GCA for 72 h in the presence or absence of 1 µM liproxstatin-1 (Lip-1) (**e**) or 1 µM CPX (**f**); ***P < 0.001. **g** Relative viability of HeLa cells following treatment with 35 nM BFA or 1.5 µM GCA for 72 h in the presence or absence of 10 µM PD-146176 (LOXi); *P < 0.05; ***P < 0.001. **h** Relative HeLa cell viability in response to 35 nM BFA for 72 h in the presence or absence of 100 µM Trolox, 4 µM NOX1 inhibitor (=GKT137831) or 10 µM pranlukast (Pran); **P < 0.01; ***P < 0.001. **c**, **e**–**h** Cell survival was determined using the CTB assay. Statistical analysis was performed using Student’s two-tailed *t*-test. Shown are representative examples of at least three independent experiments, and three wells per treatment condition were measured. Center bars indicate the mean, error bars indicate the SD. **i** Western blot analysis of HeLa cells that were treated prior to lysis with the indicated BFA or GCA concentrations for 24 h. **j** Western blot analysis of HeLa cells incubated in Cys_2_-free or low-dose (2.5 µM) Cys_2_-culture medium for 24 h before protein extraction. **d**, **i**, **j** Shown is a representative western blot of three independent experiments. Scanned images of unprocessed blots are shown in Supplementary Fig. 7

To study the effects of Golgi-disrupting compounds on cellular redox homeostasis, HeLa (Fig. 1a, b) or Jurkat T cells (Supplementary Fig. 1a) were treated with BFA or GCA, two compounds which cause Golgi dispersal and cessation of protein secretion as a consequence of GBF1 inhibition, leading to arrest of ARF G protein-controlled protein and lipid trafficking^25^. Increased levels of intracellular ROS were observed in response to both compounds in a concentration-dependent manner (see also Fig. 2a) similar to the positive control carbonyl cyanide *m*-chlorophenyl hydrazone (CCCP), an uncoupler of oxidative phosphorylation (Fig. 1a and Supplementary Fig. 1a). The tripeptide glutathione (GSH) acts as important regulator of the cellular antioxidant defense^26^, which prompted us to test whether BFA or GCA affect GSH levels. As shown in Fig. 1c, BFA and GCA treatment caused a reduction in GSH compared to vehicle-treated cells. We next assessed whether supplementation of the culture medium with GSH or another antioxidant, *N*-acetylcysteine (NAC), influences survival of HeLa cells exposed to three structurally distinct Golgi-fragmenting agents BFA, AMF-26/M-COPA^21^ and GCA^20^. Strikingly, GSH or NAC protected cells from cell death induced by these compounds (Fig. 1d). A marked right-shift of the dose response curve of HeLa cells treated for 72 h with increasing BFA concentrations was observed in the presence of a constant amount of GSH leading to an almost 10-fold higher EC50 value (Supplementary Fig. 1b). Similar effects to the ones observed in HeLa cells were detected in A549 lung adenocarcinoma or DU145 prostate cancer cells, suggesting that the induction of oxidative stress is a general occurrence in cancer cells treated with different pharmacological Golgi stressors (Supplementary Fig. 1c, d). Corroborating these results, overexpression of key enzymes involved in glutathione metabolism/homeostasis such as glutathione-disulfide reductase (GSR), which recycles GSSG to GSH, or GSTA1 increased viability of HeLa cells following BFA treatment (Fig. 1e). On the other hand, overexpression of the ROS-degrading enzymes superoxide dismutase 1 or 2 (SOD1 and SOD2), which convert superoxide (O_2_^–^) to hydrogen peroxide (H_2_O_2_), or catalase (CAT), which decomposes H_2_O_2_ to water and oxygen, did not make cells resistant to BFA treatment, suggesting that ROS species other than superoxide or hydrogen peroxide are the crucial mediators of BFA’s toxic effects **(**Supplementary Fig. 1e**)**.

### Pharmacological Golgi stress inducers trigger ferroptosis

Our observations presented in Fig. 1 hinted at the possibility that reduction of GSH levels and formation of ROS in response to BFA, GCA or AMF-26 could be directly responsible for cell death. Not long ago, a non-apoptotic, regulated cell-death pathway termed ferroptosis was described, which is closely linked to cysteine metabolism and glutathione availability. Ferroptosis is an oxidative- and iron-dependent type of cell death caused by massive lipid peroxidation that presumably causes loss of membrane integrity and ensuing cell death^6,27^. Using a probe for specific detection of lipid peroxides by fluorescence^28^, we analyzed cells in real-time following addition of BFA or GCA. The results reveal time- and concentration-dependent accumulation of lipid peroxides in HeLa cells treated with low BFA (40 or 60 nM) concentrations. For comparison, cells were treated with 10 µM erastin, a classical ferroptosis-inducing agent, which generated less lipid peroxides than BFA despite the much higher erastin concentration used (Fig. 2a). Strikingly, stable overexpression of the small G protein ARF1, which is associated with resistance to BFA and preserved Golgi structure^23,29^, caused diminished lipid peroxidation following BFA treatment in comparison to cells overexpressing an innocuous control protein (Supplementary Fig. 2a). This suggests that ARF1 is critically involved at the Golgi apparatus in regulating lipid oxidation products as a result of BFA treatment. Interestingly, loss of Arf1 in *Candida albicans* was shown to result in increased intracellular ROS accumulation^30^.

Glutathione biosynthesis can be dependent on uptake of extracellular cystine, the oxidized form of cysteine, which can be transported across the cell membrane through the heterodimeric antiporter system x_c_^−^ composed of the light chain, xCT (encoded by *SLC7A11*), and 4F2 heavy chain (4F2hc, also called CD98 and encoded by *SLC3A2*) (Fig. 2b). Since we found GSH levels to be reduced upon Golgi stress concomitant with an increase in lipid peroxidation—phenotypes that are recapitulated by treatment with the small-molecule ferroptosis inducers erastin, sorafenib, or sulfasalazine^7^—we hypothesized that BFA and GCA might elicit ferroptotic cell death. In agreement with this, simultaneous application of BFA and ferrostatin-1 (Fer-1), a potent inhibitor of ferroptosis, to a panel of cancer and primary cell lines protected cells from Golgi stress-induced cell death (Fig. 2c and Supplementary Fig. 2b). Co-treatment of cells with BFA and either Fer-1, another ferroptosis inhibitor named liproxstatin-1 (Lip-1)^31^, or GSH, ameliorated BFA-induced lipid peroxidation (Supplementary Fig. 2c). Western blot analysis of cells treated with BFA, GCA, Fer-1 or a combination thereof revealed a reduction in 78-kDa glucose-regulated protein (GRP78) (Supplementary Fig. 2d) and phospho-eIF2α expression upon Golgi stressor/Fer-1 co-treatment suggestive of attenuated ER stress (Fig. 2d). Moreover, Golgi stress-induced DNA damage as judged by H2AX phosphorylation (γH2AX) and cleaved PARP were partially suppressed in the presence of Fer-1 (Fig. 2d). The addition of Lip-1, which resembles Fer-1 by acting as a radical-trapping antioxidant^32^, to HeLa cells also made them more resistant to BFA/GCA-induced cell death (Fig. 2e). Ciclopirox olamine (CPX), an intracellular iron chelator, likewise partially protected HeLa cells from lethality caused by BFA or AMF-26 (Fig. 2f and Supplementary Fig. 2e) and to a certain degree reduced BFA/GCA-stimulated GRP78 expression and cleaved PARP similar to Lip-1 treatment, GSH addition or pharmacological nicotinamide adenine dinucleotide phosphate (NADPH) oxidase (NOX) inhibition using GKT137831 (NOX1/4 inhibitor) (Supplementary Fig. 2f). Cell death induced by another Golgi-dispersing compound, AG1478/tyrphostin^33^, was also attenuated in the presence of Fer-1 or Lip-1 (Supplementary Fig. 2g). Lipoxygenases, which use molecular oxygen to form hydroperoxyl groups at different carbon positions of acyl chains, were previously shown as important enzymatic contributors to accumulation of lipid peroxides in ferroptosis^34,35^. Co-treatment of HeLa cells with BFA or GCA and PD-146176 (LOXi), a selective inhibitor of arachidonate 15-lipoxygenase (ALOX15), was sufficient to increase cell viability compared to BFA- or GCA-single treatments (Fig. 2g). Another scavenger compound with the ability to counterbalance ferroptosis is the antioxidant trolox, which also increased the survival of BFA-treated cells (Fig. 2h). The NADPH oxidases (NOXs), a family of enzymes that transfers electrons from NADPH inside cells across the membrane and couple these to molecular oxygen, thereby producing superoxide from oxygen, were previously implicated in playing critical roles in ferroptosis in certain cancer cell lines^6^. Substantiating a key role for ferroptosis in response to Golgi stressors, we observed that co-incubation of cells with GKT137831, a NOX1/4 inhibitor (NOXi), in combination with AMF-26 or BFA partially rescued cells treated with Golgi stress-inducing compounds in HeLa (Fig. 2h and Supplementary Fig. 2e, 2f) or MDA-MB-231 cells (Supplementary Fig. 2h). These results were furthermore validated by lentiviral-mediated knockdown studies using shRNAs against NOX1, which made HeLa cells resistant to BFA treatment (Supplementary Fig. 2i). In line with a previous report, the leukotriene C_4_ receptor (CysLTR1) antagonist pranlukast (Pran) strongly protected HeLa cells against AMF-26 or BFA (Fig. 2h and Supplementary Fig. 2e) presumably by inhibiting NOX4-induced ROS generation^36^. It was previously found that NOX1 can interact with dipeptidyl-peptidase-4 (DPP4) to promote lipid peroxidation/ferroptosis^37^. Consistent with this, we found that vildagliptin, a DPP4 inhibitor, made cells more resistant to the cytotoxic effects of BFA or GCA (Supplementary Fig. 2j, 2k). Although the effects of the tumor suppressor p53 on cellular sensitivity to ferroptosis are controversial^37–40^, recent evidence suggests that p53 stabilization can restrain the induction of ferroptosis due to slower glutathione depletion and decreased lipid ROS formation^37,38^. We therefore also tested in the absence or presence of BFA the effects on cellular survival of nutlin-3, which inhibits the interaction of MDM2 and p53 to cause stabilization of the latter. HeLa cells co-treated with nutlin-3 and BFA displayed an increased survival ratio compared with single BFA treatment (Supplementary Fig. 2l). Similarly, nutlin-3 treatment protected A549 cells from GCA-induced toxicity (Supplementary Fig. 2m). Co-treatment of cells with tunicamycin (TM), an ER stress-inducing *N*-glycosylation inhibitor, and Fer-1 did not protect cells from undergoing TM-induced cell death (Supplementary Fig. 2n), suggesting that ferroptosis is not a general feature associated with compounds that trigger the unfolded protein response. Finally, we found that BFA/GCA treatment or cysteine-deprivation caused a qualitatively similar regulation of factors involved in ferroptosis signaling including an induction of the two presumptive erastin targets, SLC7A11 and SLC7A5^6,41^, and a reduction in ACSL4 protein levels, suggesting that both conditions stimulate at least partially overlapping downstream signaling mechanisms (Fig. 2i, j). This is also congruent with a transcriptomic analysis of erastin-treated cells demonstrating that erastin triggers ER stress^41^ as does BFA^23^.

### Ferroptosis inhibition protects from BFA-induced Golgi dispersal

**Fig. 3:**
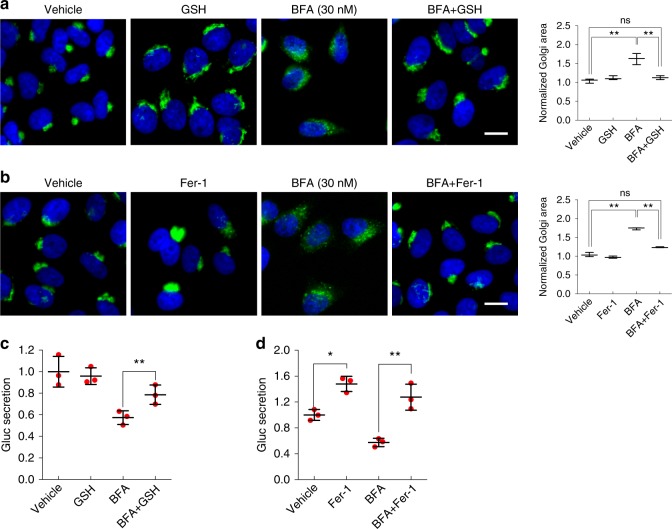
Impact of ferroptosis inhibitors on Golgi morphology and protein secretion. **a** Immunofluorescence microscopic pictures of HeLa cells that were either vehicle-treated, treated with 30 nM BFA, 2 mM GSH or a combination thereof for 72 h before fixation and staining for the *cis*-Golgi marker GM130; scale bar: 20 µm. Quantification of Golgi area measurements is shown in the bar graphs on the right; **P < 0.01. **b** Immunofluorescence microscopic pictures of HeLa cells treated for 72 h with vehicle, 35 nM BFA, 10 µM ferrostatin-1 (Fer-1), or a combination thereof before fixation and staining for GM130; scale bar: 20 µm. Graphs on the right display the quantification of Golgi area measurements shown in the immunofluorescence images; **P < 0.01. Center bars indicate the median, and tails indicate the minimum and maximum. **a**, **b** Shown are representative images of two independent experiments each time quantifying Golgi area measurements of approximately 1000 cells derived from three wells per genotype and condition. **c**, **d** HeLa stably overexpressing *Gaussia* Luciferase (Gluc-flag) treated with 40 nM BFA alone or in combination with 2 mM GSH (**c**) or 10 μM Fer-1 (**d**) for 2 h. Before BFA addition, cells were pretreated for 24 with GSH or Fer-1, respectively. The secretion was determined as a ratio calculated by dividing the luminescence values of treated samples by the values of the corresponding vehicle control (after signal background subtraction). **c**, **d** Center bars indicate the mean, error bars indicate the SD. Shown is a representative example of two independent experiments each time measuring three wells per genotype and condition; *P < 0.05, **P < 0.01 (Student’s two-tailed test)

We next analyzed by immunofluorescence microscopy Golgi morphology of cells exposed to BFA, GSH, Fer-1 or to the combination of BFA with either GSH or Fer-1. Immunofluorescence staining for the *cis*-Golgi marker GM130 revealed that BFA-induced Golgi dispersal was substantially diminished upon simultaneous GSH exposure leading to complete restoration of the measured Golgi area to baseline levels of untreated cells (Fig. 3a). Akin to the results obtained with GSH, a striking rescue of Golgi scattering in cells co-treated with BFA and Fer-1 was detected compared to BFA-only treatment (Fig. 3b). Furthermore, co-treatment of BFA with GSH and Fer-1, respectively, improved secretion of a reporter protein (*Gaussia* luciferase, Gluc) relative to BFA-only treatment (Fig. 3c, d). Interestingly, Fer-1 by itself appeared to promote protein secretion (Fig. 3d). Together, these data not only demonstrate a key role for ferroptosis in governing Golgi stress-triggered cell death, but also suggest that reduced accumulation of lipid peroxides rectifies Golgi dispersal as well as protein secretion in response to AMF-26, BFA or GCA.

### Golgi stress and key ferroptosis components

**Fig. 4:**
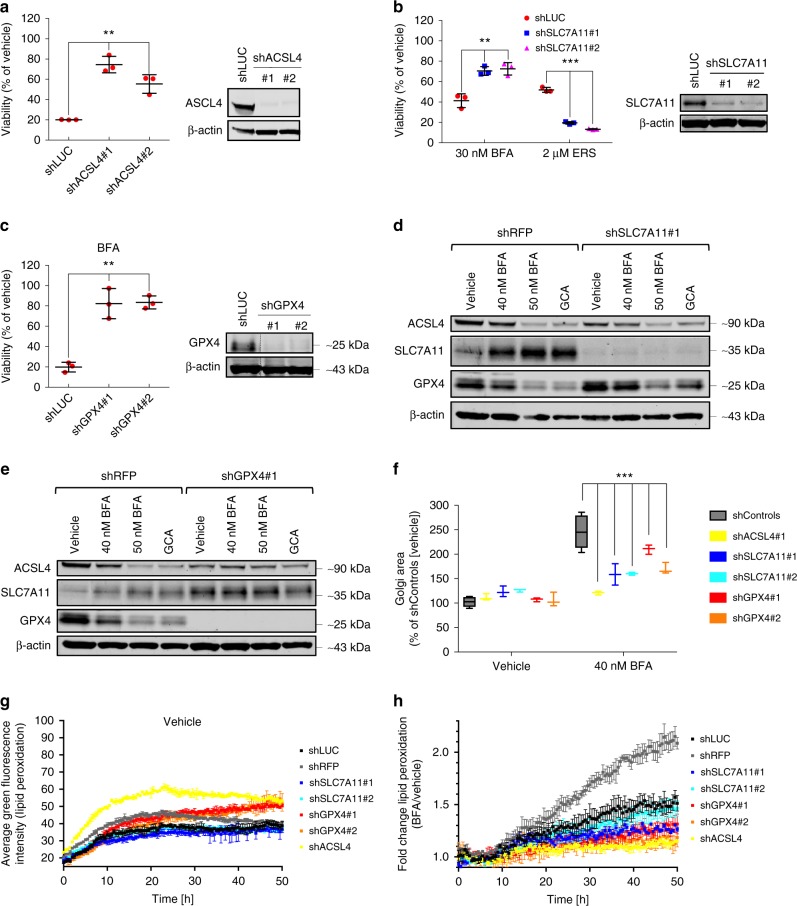
Loss of function studies of key ferroptosis regulators in the context of Golgi stress. **a** Relative survival of control (shLUC) and ACSL4 knockdown HeLa cells following treatment with 35 nM BFA for 72 h; **P < 0.01. ACSL4 knockdown levels are shown by immunoblotting. **b** Survival ratios of SLC7A11 knockdown and control HeLa cells after treatment for 72 h with either 30 nM BFA or 2 µM erastin; **P < 0.01; ***P < 0.001. SLC7A11 expression is shown by immunoblot analysis. **c** Relative viability of GPX4 knockdown or control HeLa cells after treatment with 35 nM BFA for 72 h; **P < 0.01. Expression levels of GPX4 are shown by western blot. Protein lysates were run on the same gel, and dashed lines in blots indicate where irrelevant samples were cropped out. **a**–**c** Cell survival of vehicle-treated and BFA-treated cells was analyzed using the CTB assay. Statistical analysis was performed using Student’s two-tailed *t*-test. Shown are representative examples of three independent experiments, and three wells per genotype and treatment condition were measured. Center bars indicate the mean, error bars indicate the SD. **d** Representative immunoblot analysis (*n* = 2) of HeLa SLC7A11 knockdown (shSLC7A11#1) or control (shRFP) cells treated for 72 h with either vehicle, 40 nM and 50 nM BFA or 1.5 μM GCA. **e** Representative immunoblot analysis (*n* = 2) of GPX4-depleted (shGPX4#1) or control HeLa cells treated for 72 h with vehicle, 40 nM and 50 nM BFA or 1.5μM GCA. Note that the same shRFP protein lysates were loaded on separate gels in **d** and **e**. **f** HeLa knockdown cells with the indicated genotypes were treated for 72 h with 40 nM BFA before fixation, GM130 immunofluorescence staining and image acquisition. Graph shows quantification of Golgi area following vehicle or BFA treatment. Center bars indicate the median, and tails indicate the minimum and maximum; boxes extend from the 25th to 75th percentiles. shControls refers to shLUC and shRFP hairpins which were combined for the analysis. On average 1000 cells were analyzed for Golgi area quantification; ***P < 0.001. The corresponding immunofluorescence pictures are presented in Supplementary Fig. 4. **g** Real-time lipid peroxidation measurements using Liperfluo dye added to vehicle-treated HeLa knockdown cells of the genotypes shown for the indicated amount of time. Each point presented is the average fluorescence intensity derived from three separate wells, and four pictures per well were measured. Error bars indicate SD of the mean. **h** Relative lipid peroxide fold changes as calculated by dividing the average GFP values (derived from three wells) of HeLa knockdown cells of the indicated genotype treated with 40 nM BFA by their corresponding average GFP values of vehicle-treated cells. Error bars indicate SD of the mean. Statistical significance between the different treatment conditions was calculated using a two-way ANOVA test. GPX4 and SLC7A11 knockdown cell lines (at least one of the two hairpins targeting either GPX4 or SLC7A11) displayed significantly reduced lipid peroxide-fold changes compared to both shRFP and shLUC control knockdown cell lines starting approximately at 24 h (GPX4 knockdown cells) or 30 h (SLC7A11 knockdown cells) of BFA treatment (in comparison to shRFP control cells, significance is reached earlier); *P* < 0.05. Scanned images of unprocessed blots are shown in Supplementary Fig. 7

Overexpression of SLC7A11 or GPX4-cyto was previously reported to confer protection to erastin-induced ferroptosis^6,27^. In line with this and our own results from above, we found that stable overexpression of epitope-tagged SLC7A11 (Supplementary Fig. 3a) or GPX4-mito (Supplementary Fig. 3b) made HeLa cells more resistant to Golgi stressors. Moreover, treatment of HeLa cells with BFA impaired in vitro colony formation, an effect which was attenuated when GPX4 was overexpressed (Supplementary Fig. 3c). Recently, ACSL4 was shown to be essential for ferroptotic cell death as its knockdown provided marked resistance to ferroptosis-inducing agents^13,14,42^. Recapitulating these effects, we observed that stable ACSL4 HeLa knockdown cell lines became resistant to BFA treatment (Fig. 4a). Conversely, ACSL4 overexpression sensitized HeLa cells to BFA treatment (Supplementary Fig. 3d). In addition to the GSH-glutaredoxin antioxidant pathway, the cellular redox environment is also controlled by the thioredoxin system, the latter one including thioredoxin reductase 1 (TXNRD1). Crosstalk and functional compensation has been observed between these two systems^43–45^. Lentiviral-mediated knockdown of TXNRD1-sensitized cells to cell death in the presence of BFA (Supplementary Fig. 3e). Similarly, co-incubation of HeLa cells with the thioredoxin inhibitor Auranofin and BFA caused a further decrease in viability relative to single BFA treatment (Supplementary Fig. 3f). This suggests that redox balance in response to Golgi stress-inducing compounds is regulated by both antioxidant systems. Despite the observation by Tang and colleagues that activation of the KEAP1-NRF2 pathway protects hepatocellular carcinoma cells against ferroptosis^46^, in our hands knockdown of NRF2 in HeLa cells did not show obvious alterations in their sensitivity towards BFA relative to control knockdown cells (Supplementary Fig. 3g). Intriguingly, lentiviral hairpin-mediated SLC7A11 or GPX4 depletion markedly increased cellular resistance to BFA (Fig. 4b, c). This result was unexpected, because SLC7A11 or GPX4 gain-of-function prevent BFA-induced cell death (Supplementary Fig. 3a, 3b), *Gpx4* deficiency by itself was shown to entail ferroptosis^31^, and RNAi-mediated GPX4 or SLC7A11 knockdown sensitized to RSL3- or erastin-induced ferroptosis^6,27^, and hence a similar sensitization phenotype could have been expected in the presence of BFA. To confirm the previously described ferroptosis sensitization effects, we treated the SLC7A11 knockdown and control cells concurrently with either BFA or erastin. We observed the anticipated erastin sensitivity phenotype suggesting that BFA-resistance upon SLC7A11 depletion in our system is not due to hairpin-off target effects (Fig. 4b). Strikingly, when lysates of SLC7A11- or GPX4-depleted HeLa cells were analyzed, which prior to lysis were left untreated or treated with BFA or GCA, we found ACSL4 expression to be concomitantly reduced in SLC7A11 or GPX4 knockdown cells relative to shRFP control cells (Fig. 4d, e). Similar to BFA/GCA treatment, it was previously reported that erastin suppresses GPX4 protein expression^47^. Of note, GPX4 expression was slightly enhanced in SLC7A11 knockdown cells and became to a lesser extent downregulated in response to BFA or GCA relative to control cells (Fig. 4d). On the other hand, SLC7A11 expression was induced following GPX4 downregulation (Fig. 4e). These effects are likely to contribute to enhanced BFA/GCA resistance in the respective knockdown cells. Since loss of ACSL4 renders cells resistant to BFA-induced cell death (Fig. 4a), it is conceivable that the observed ACSL4 downregulation as a result of shRNA-induced SLC7A11 or GPX4 knockdown is epistatic to loss of SLC7A11 or GPX4. In other words, in SLC7A11- or GPX4-depleted HeLa cells BFA-induced ferroptosis progression is attenuated due to a concomitant reduction of ACSL4 levels. In a similar vein, Doll et al.^13^ demonstrated that lipid oxidation caused by GPX4 inhibition requires ACSL4.

We previously reported that genetic or pharmacological downregulation of autophagy enhances survival of cells exposed to BFA^24^. Hence, by immunoblotting we also tested the effects of ACSL4, SLC7A11 and GPX4 knockdown on autophagy flux as assessed by LC3-II formation. Using two independent hairpins targeting either of the three ferroptosis-associated factors, it became evident that LC3-II levels were reduced in ACSL4-, GPX4- or SLC7A11-depleted cells both in the absence and presence of bafilomycin A (Supplementary Fig. 3h–j). While we did not explore additional approaches to more comprehensively characterize the extent of autophagy modulation, these results are nonetheless in agreement with the notion that attenuated autophagy flux of cells depleted of ACSL4, GPX4, or SLC7A11 might be a contributing factor to their increased resistance to BFA. Congruent with their enhanced viability following Golgi stress, ACSL4-, SLC7A11-, and GPX4 knockdown cells displayed a substantially reduced Golgi dispersal in response to BFA treatment compared to control cells (Fig. 4f and Supplementary Fig. 4). Last, we measured lipid peroxide formation in HeLa cells transduced with lentiviral hairpins against ACSL4, SLC7A11, GPX4 or LUC and RFP as controls **(**Fig. 4g**)**. The two independent knockdown cell lines for either SLC7A11 or GPX4 showed baseline lipid peroxidation levels comparable to shLUC or shRFP control cells, suggesting that depletion of either ferroptosis factor does not induce overt oxidative lipid degradation in our experimental setup (Fig. 4g). In addition, BFA treatment caused less relative lipid peroxide formation in SLC7A11- and GPX4-depleted cells than in control cells (Fig. 4h). Interestingly, the single ACSL4 knockdown line we tested displayed slightly higher Liperfluo staining under vehicle-treated conditions compared to shLUC and shRFP cells (Fig. 4g). However, as expected, BFA-induced lipid peroxidation staining was greatly diminished in comparison to control knockdown cell lines (Fig. 4h).

### Effects of low doses of ferroptosis inducers and Golgi stressors

**Fig. 5:**
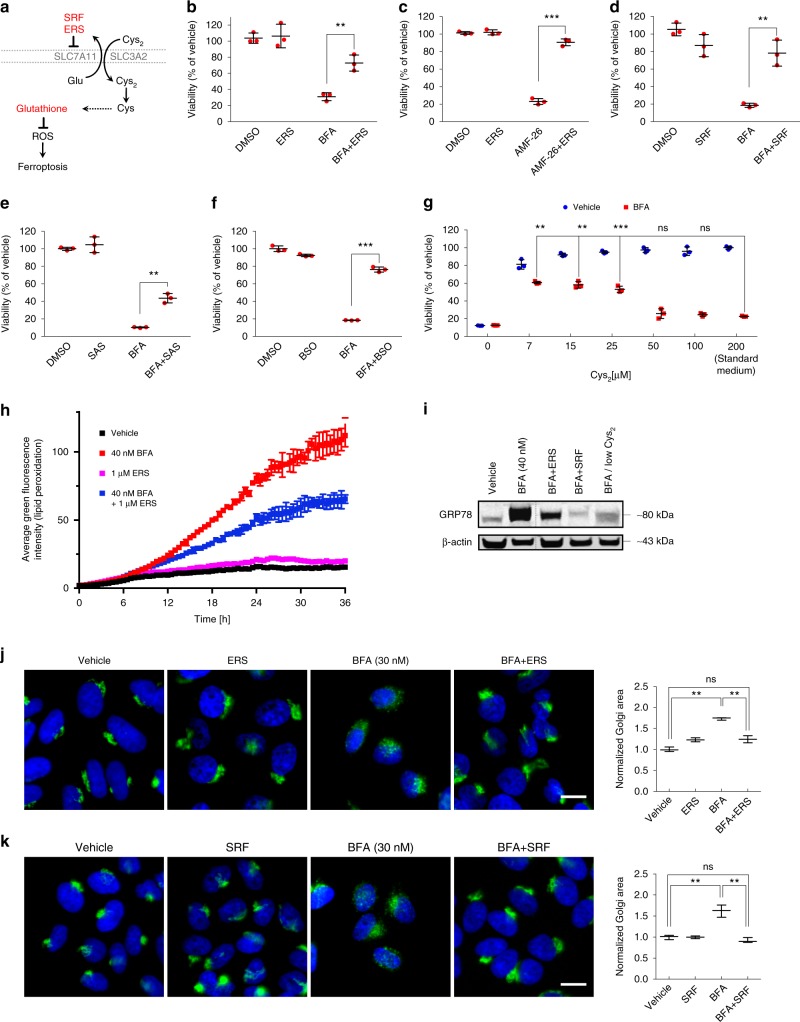
Cellular responses to low doses of ferroptosis inducers. **a** Schematic illustration of Cys_2_ uptake and GSH metabolism including known ferroptosis inducers such as erastin (ERS) and sorafenib (SRF). **b**–**f** Relative viability of HeLa cells treated with with BFA (35 nM) or AMF-26 (35 nM) for 72 h in the presence or absence of sublethal doses of erastin (ERS, 1 µM), sorafenib (SRF, 1 µM), sulfasalazine (SAS, 200 µM), L-buthionine sulfoximine (BSO, 50 µM); **P < 0.01; ***P < 0.001. **g** Shown is the relative survival of HeLa cells grown in medium supplemented with increasing concentrations of Cys_2_ for 72 h in the presence or absence of 35 nM BFA; **P < 0.01; ***P < 0.001. **b**–**g** Statistical analysis was performed using Student’s two-tailed *t*-test. Shown are representative examples of three independent experiments, and three wells per treatment condition were measured. Center bars indicate the mean, error bars indicate the SD. **h** Real-time analysis of lipid peroxide formation of HeLa cells following BFA treatment in the presence or absence of 1 µM erastin (ERS) using Liperfluo staining. Shown is a representative example of two independent experiments each time measuring the average fluorescence intensity which was derived from four pictures taken per well from five wells. Statistical significance between the different treatment conditions was calculated using a two-way ANOVA test. [BFA + 1 μM ERS] treatment caused significant lower lipid peroxide generation compared to BFA-only treatment starting at 13 h of treatment, whereas 1 μM ERS by itself had no significant effect on oxidation of lipids; *P* < 0.01. **i** HeLa cells were treated for 72 h with 40 nM BFA in the presence or absence of ferrostatin-1 (Fer-1, 10 µM), low concentration of erastin (ERS, 1 µM), sorafenib (SRF, 1 µM) or medium containing 7 µM Cys_2_ (low Cys_2_) for 72 h. Shown is a representative western blot of three independent experiments. Protein lysates were run on the same gel, and dashed lines in scans indicate where irrelevant samples were cropped out. **j** Immunofluorescence microscopic pictures of HeLa cells treated for 72 h with vehicle, 1 µM erastin (ERS), 30 nM BFA or a combination thereof; scale bar: 20 µm. **k** Immunofluorescence microscopic pictures of HeLa cells vehicle-treated, treated with 30 nM BFA, 1 µM sorafenib or a combination thereof for 72 h; scale bar: 20 µm. **j**, **k** Quantification of Golgi areas of HeLa cells depicted in the immunofluorescence images on the left is shown in the right graphs. Center bars indicate the median, and tails indicate the minimum and maximum. On average 1000 cells per genotype and conditions were analyzed for the quantification; **P < 0.01. **b**–**g** Cell survival was determined using the CTB assay. Scanned images of unprocessed blots are shown in Supplementary Fig. 7

Having established that inhibition of ferroptosis protects from small molecule-induced Golgi dispersal and lethality, we next tested the effects of several established ferroptosis inducers such as erastin, sorafenib, sulfasalazine or RSL3 (Fig. 5a) in conjunction with Golgi stressors. Because ferroptosis-inducing agents cause overt cellular toxicity at concentrations commonly used in the literature, for our chronic survival assays we applied lower doses of these compounds. Surprisingly, using sublethal concentrations of erastin, sorafenib or sulfasalazine in combination with AMF-26 or BFA, we found that these agents strongly diminished the cell death-inducing effect of BFA (Fig. 5b, d, e) or AMF-26 (Fig. 5c). Additionally, HeLa cells co-treated with a low erastin dose and BFA were capable of forming colonies in clonogenic assays unlike cells treated with BFA alone (Supplementary Fig. 5a). Moreover, buthionine sulfoximine (BSO), a specific inhibitor of glutamate-cysteine ligase (also known as γ-glutamylcysteine synthetase), the first rate-limiting enzyme of GSH synthesis, was also able to protect cells from BFA-mediated lethality (Fig. 5f). On the other hand, cell death resulting from a higher erastin concentration could not be blocked by a low BFA dose (Supplementary Fig. 5b). A sublethal RSL3 dosage did not rescue cells from BFA toxicity (Supplementary Fig. 5c). The rather unexpected rescuing effect of nontoxic erastin treatment was also recapitulated in A549 cells (Supplementary Fig. 5d). Unlike AMF-26 or BFA, treatment of HeLa cells with either cisplatin or doxorubicin in combination with 1 µM erastin did not rescue cells from DNA damage-triggered cell death (Supplementary Fig. 5e, 5f).

The effects of erastin, which inhibits SLC7A11-mediated cystine import, can be mimicked by a reduction in the amount of extracellular cystine levels resulting in reduced levels of intracellular cysteine, which is required for GSH synthesis^48^. Accordingly, we tested whether a decrease in Cys_2_ levels in the cell culture medium could phenocopy the effects of erastin and other ferroptosis inducers described above. Indeed, lowering the Cys_2_ concentration from the standard 200 µM in the medium to a range between 5 and 25 µM, enhanced cellular resistance to BFA (Fig. 5g). To assess the levels of oxidative degradation of lipids upon exposure of HeLa cells to a sublethal erastin concentration, cells were monitored using the IncuCyte live-cell analysis system following addition of a lipid peroxide-detecting probe as described above. Unlike cells treated with 10 µM erastin (Fig. 2a), no lipid peroxide induction in cells exposed to 1 µM erastin was detected. On the contrary, whereas 1 µM erastin treatment had no effect by itself, it nonetheless negated BFA-induced lipid peroxidation (Fig. 5h). By immunoblotting we found that BFA-induced ER stress induction was attenuated upon co-treatment of BFA with sublethal doses of ferroptosis inducers or BFA treatment in low Cys_2_ culture media as judged by GRP78 expression (Fig. 5i). Complementing above findings, immunostaining for the *cis*-Golgi marker GM130 revealed that while 1 µM erastin alone did not affect Golgi morphology, it strongly protected HeLa cells from BFA-induced Golgi dispersal (Fig. 5j). A similar protective effect on Golgi structure was observed with a sublethal sorafenib dose (Fig. 5k). Moreover, treatment with 1 µM erastin but not sorafenib increased the secretory capacity of cells in the presence of BFA compared to single BFA treatment (Supplementary Fig. 5g). Intriguingly, ACSL4 levels were reduced, while at the same time SLC7A11 expression was slightly elevated relative to vehicle-treated cells (Supplementary Fig. 5h). Together, the observed findings using nontoxic doses of ferroptosis inducers or low Cys_2_ conditions might be related to co-regulatory effects and feedback mechanisms induced by these conditions through priming the cellular antioxidant defense, which could result in increased cellular capacity to resist BFA-triggered ferroptosis through mitigation of lipid peroxidation.

**Table 1:** Cloning of cDNAs and hairpin IDs

Gene	Forward primer	Reverse primer	Vector	cDNA source
*GSR NM_000637.3_var1*	*GSR/SalI*	*GSR/NotI*	*pLJM60*	PC3
*GSTA1(var2-3)*	*GSTA1_var2/SalI*	*GSTA1/NotI*	*pLJM60*	A549
*GPX4_var1_mito* *NM_002085.4*	*GPX4/AgeI*	*GPX4-flag/EcoRI*	*pLJM13*	HeLa
*SLC7A11* *NM_014331.3*	*SLC7A11/AgeI*	*SLC7A11-flag/EcoRI*	*pLJM13*	A549
*ACSL4* *NM_001318509*	*ACSL4var3/SalI*	*ACSL4/NotI*	*pLJM60*	HeLa
*SOD1 (cytoplasm.)* *NM_000454.4*	*SOD1/AgeI*	*SOD1-flag/EcoRI*	*pLJM13*	A549
*SOD2 (mitoch.)* *NM_000636.3*	*SOD2/AgeI*	*SOD2-flag/EcoRI*	*pLJM13*	A549
*CAT* *NM_001752.3*	*CAT/AgeI*	*CAT-flag/XbaI*	*pLJM13*	A549

The cloning of Flag-γ-Tubulin was described previously^81^. For cloning of all other cDNAs described in the manuscript, the above oligos and cDNA pools were used for the PCR reactions

**Table 2:** *GSTA1(var2-3)* cDNA sequence

*ATG* *TATATAGAAGGTATAGCAGATTTGGGTGAAATGATCCTCCTTCTGCCCGTATGTCCACCTGAGGAAAAAGATGCCAAGCTTGCCTTGATCAAGGAGAAAATAAAAAATCGCTACTTCCCTGCCTTTGAAAAAGTCTTAAAGAGCCATGGACAAGACTACCTTGTTGGCAACAAGCTGAGCCGGGCTGACATTCATCTGGT* *GGAACTTCTCTACTACGTCGAGGAGCTTGACTCCAGTCTTATCTCCAGCTTCCCTCTGCTGAAGGCCCTGAAAACCAGAATCAGCAACCTGCCCACAGTGAAGAAGTTTCTACAGCCTGGCAGCCCAAGGAAGCCTCCCATGGATGAGAAATCTTTAGAAGAAGCAAGGAAGATTTTCAGGTTT* *TAA*	

Several polymorphisms in *GSTA1* exist. Shown above is the cDNA sequence cloned into our lentiviral vectors for overexpression studies (shown is only the ORF). Underlined sections indicate the start and stop codons, respectively.

**Table 3:** Primer sequences used for PCR-amplification of cDNAs

*GSR/SalI: GTCGACcATGGCCCTGCTGCCCCGAGC*
*GSR/NotI: gcggccgcTCAACGAAGTGTGACCAGC*
*GSTA1_var2/SalI: GTCGACtATGTATATAGAAGGTATAGC*
*GSTA1/NotI: gcggccgcTTAAAACCTGAAAATCTTCC*
*GPX4/AgeI: accggtATGAGCCTCGGCCGCCTTTGC*
*GPX4-flag/EcoRI: gaattcTCActtgtcatcgtcatccttgtaatcGAAATAGTGGGGCAGGTCC*
*SLC7A11/AgeI: accggtATGGTCAGAAAGCCTGTTGTG*
*SLC7A11-flag/EcoRI: gaattcTCActtgtcatcgtcatccttgtaatcTAACTTATCTTCTTCTGGTAC*
*ACSL4var3/SalI: GTCGACtATGAAACTTAAGCTAAATGTGC*
*ACSL4/NotI: gcggccgcTTATTTGCCCCCATACATTCG*
*SOD1/AgeI: accggtATGGCGACGAAGGCCGTGTGC*
*SOD1-flag/EcoRI: gaattcTCActtgtcatcgtcatccttgtaatcTTGGGCGATCCCAATTACACC*
*SOD2/AgeI: accggtATGTTGAGCCGGGCAGTGTGC*
*SOD2-flag/EcoRI: gaattcTCActtgtcatcgtcatccttgtaatcCTTTTTGCAAGCCATGTATC*
*CAT/AgeI: accggtATGGCTGACAGCCGGGATCC*
*CAT-flag/XbaI: tctagaTCActtgtcatcgtcatccttgtaatcCAGATTTGCCTTCTCCCTTGC*

**Table 4:** Mission shRNAs (Sigma-Aldrich) used for knockdown studies (TRC ID)

shACSL4#1: TRCN0000045539
shACSL4#2: TRCN0000045540
shSLC7A11#1: TRCN0000043124 (HeLa)
shSLC7A11#2: TRCN0000288866 (HeLa)
shSLC7A11#1: TRCN0000288926 (A549 & PANC1)
shSLC7A11#2: TRCN0000043124 (A549 & PANC1)
shSLC7A11#3: TRCN0000288866 (A549 & PANC1)
shGPX4#1: TRCN0000046251
shGPX4#2: TRCN0000300410
shNOX1#1: TRCN0000428100
shNOX1#2: TRCN0000415324
shTXNRD1#1: TRCN0000046534
shTXNRD1#2: TRCN0000046533
shNRF2 (NFE2L2)#1: TRCN0000007558
shNRF2 (NFE2L2)#2: TRCN0000284999

### The transsulfuration pathway and Golgi stress

**Fig. 6:**
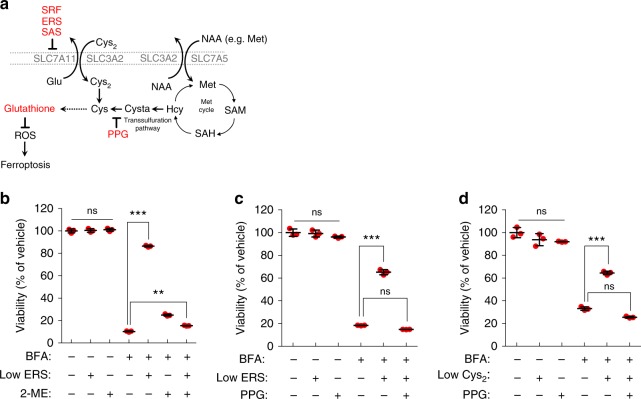
Pharmacological inhibition of the transsulfuration pathway counteracts the effects of sublethal erastin doses or cystine-deprived conditions in the presence of BFA. **a** Schematic illustration of Cys_2_ uptake/metabolism, GSH biosynthesis and the transsulfuration pathway. Propargylglycine (PPG) is a cystathionine-γ-lyase (CTH) inhibitor. **b** Relative viability of HeLa cells following treatment with 35 nM BFA and 1 µM ERS for 72 h in the presence or absence of 50 µM 2-Mercaptoethanol (2-ME); **P < 0.01, ***P < 0.001. **c** Relative viability of HeLa cells following treatment with 35 nM BFA and 1 µM ERS for 72 h in the presence or absence of 1 mM PPG, ***P < 0.001. **d** Relative viability of HeLa cells after treatment with 35 nM BFA and 1 mM PPG for 72 h in normal medium or medium with a low Cys_2_ concentration (7 µM); ***P < 0.01. **b**–**d** Cell survival was measured by the CTB assay; statistical analysis was performed using a one-way ANOVA test. Shown are representative examples of three independent experiments, and three wells per genotype and treatment condition were measured. Center bars indicate the mean, error bars indicate the SD

β-mercaptoethanol/2-mercaptoethanol (β-ME/2-ME) reacts with cystine in the culture medium to generate mixed disulfides, which subsequently can be taken up by other transporters such as system L (SLC3A2/SLC7A5), a transport system for neutral α-amino acids, instead of system x_c_^−^ (Fig. 6a)^49^. Stockwell and colleagues previously observed that ferroptosis induction by erastin was strongly inhibited when cells were co-incubated with 2-ME^6^. Strikingly, 2-ME abolished to a large extent the rescuing effect of low dose erastin treatment concurrently exposed to BFA (Fig. 6b). Since ferroptosis induction by BFA depends on the availability of extracellular cystine (Fig. 5g), we reasoned that intracellular sources of cysteine might also play important roles when cells are facing Golgi stress conditions or extracellular Cys_2_ limitation. The transsulfuration pathway (Fig. 6a) can act as a resistance mechanism against ferroptosis^50^. Using propargylglycine (PPG), we evaluated whether pharmacological inhibition of cystathionine γ-lyase, an enzyme converting cystathionine to cysteine as the last step of the transsulfuration pathway, modulates sublethal erastin-mediated effects on survival following Golgi stress. As shown in Fig. 6c, PPG effectively prevented the rescuing effect of nontoxic erastin treatment on BFA-mediated toxicity. Furthermore, application of PPG to HeLa cells cultured in low Cys_2_ culture medium in the presence of BFA led to their re-sensitization and caused a similar survival as BFA-only treatment (Fig. 6d). Together, these results underscore the importance of the transsulfuration pathway to enhance cell viability following treatment with low concentrations of ferroptosis inducers in the presence of BFA or culturing cells in a low Cys_2_ environment.

## Discussion

We and others previously reported that the Golgi disruptor BFA promotes apoptotic cell death. However, treatment with a pan caspase inhibitor only partially rescued survival of BFA-treated cells suggesting the engagement of additional cell death modalities^24,51^. In this study, we have identified ferroptosis as an important cell death pathway in response to several Golgi-disrupting compounds such as AMF-26, BFA, GCA, and AG1478/tyrphostin. We have demonstrated that a diverse panel of ferroptosis-modulating compounds have protective effects on Golgi morphology and functionality following treatment with the above Golgi stressors. Several seemingly counterintuitive observations following concurrent treatment of cells with Golgi stressors and different ferroptosis inducers, the latter used at sublethal concentrations were made: cells became protected from undergoing cell death due to BFA toxicity, BFA-induced Golgi-dispersal was prevented, and inhibition of protein secretion provoked by BFA was counteracted. The simultaneous application of a nontoxic erastin dose to cells was sufficient to alleviate Golgi stress-induced lipid peroxidation. The transsulfuration pathway may act as a compensatory system for cysteine provision following oxidative challenge as a mechanism to limit ferroptosis^11,50^. Along these lines, we found that the pro-survival effects of a low dose of erastin in combination with Golgi stressors were abrogated when concurrently applied to cells with a pharmacological inhibitor of the transsulfuration pathway.

Similarly unanticipated and perhaps mechanistically related to above findings are our results revealing that shRNA-mediated SLC7A11 or GPX4 knockdown cells were largely resistant to BFA treatment. Because the BFA-resistant SLC7A11 knockdown cells are sensitive to erastin, which was also previously shown by others^6^, it is conceivable that alterations in cellular metabolism, signaling pathways and/or cell death components caused by SLC7A11 downregulation differentially impact the response to BFA and erastin whose mechanisms of action are distinct. Intriguingly, sublethal lipid peroxidation products including 4-hydroxynonenal were shown to protect from oxidative stress^52,53^. Cells with reduced SLC7A11 or GPX4 expression or treated with nontoxic doses of ferroptosis-inducing agents might experience a low level oxidative stress situation that could lead to a minor disruption of ROS homeostasis. This in turn might trigger a compensatory, adaptive cellular response including modulation of the transsulfuration pathway able to thwart further lipid ROS damage induced by Golgi stressors. Furthermore, treatment with sublethal concentrations of several ferroptosis-inducing agents, low cystine growth conditions or lentiviral hairpin-mediated depletion of SLC7A11 or GPX4 was associated with ACSL4 downregulation; additionally we found that sublethal concentrations of ferroptosis inducers increased SLC7A5 and SLC7A11 expression, which could help cells to better cope with oxidative stress^34^. Lastly, downregulation of ACSL4, SLC7A11 or GPX4 appears to attenuate autophagy induction, which further protects from BFA-induced toxicity^24^. These results are in line with recent observations that ferroptosis is promoted by cargo-specific autophagy known as ferritinophagy, which enhances iron supply for ferroptotic cell death^54^.

It was reported that toxic lipid peroxidation products accumulate in ER-associated compartments^34^, and that 4-hydroxynonenal as well as other aldehydes or lipid intermediates are able to inhibit Golgi glycosyltransferases^55^. Moreover, a query of the human protein atlas (http://www.proteinatlas.org) indicates that in several cancer cell lines ACSL4 localizes to the Golgi apparatus. Given ACSL4’s role as critical determinant of ferroptosis sensitivity, this suggests that lethal lipid ROS species might also be generated in Golgi membranes from where the signal could be propagated to other compartments. Interestingly, findings from a rat brain ischemia/reperfusion model suggest a causal relationship between the accumulation of lipid peroxidation products in the Golgi and perturbations to its ultrastructure^56^. Several neurodegenerative disorders including Alzheimer’s and Parkinsons’s are associated not only with Golgi fragmentation but also with oxidative stress/lipid peroxide generation^57,58^. Additionally, oxidative stress and Golgi disruption have been linked also in other instances, but the cause-effect relationship is currently not established^59^.

Our findings presented here furthermore raise a more general question, namely whether or not the formation of lipid-based ROS is an obligatory consequence of pharmacologically induced Golgi dispersal regardless of the precise mechanism of action of the compound. To address this point, we decided to treat cells with additional small molecules that perturb Golgi structure, such as doxorubicin^60,61^, panobinostat^61^, or nocodazole^61,62^ at concentrations previously shown to elicit Golgi disruption, and measured the generation of lipid peroxides. Doxorubicin at both and nocodazole at the higher concentration indeed caused lipid peroxide accumulation. On the other hand, panobinostat used at low nanomolar concentrations did not evoke a similar response, which, however, does not exclude the possibility that higher panobinostat concentrations could entail lipid peroxidation^63^ (Supplementary Fig. 6). A more detailed analysis including the use of genetic Golgi dispersal models will be invaluable to clarify this issue. Assuming lipid ROS formation is intrinsic to Golgi fragmentation, what might be the underlying molecular basis? It is conceivable that Golgi dispersal could compromise cellular stress resistance, possibly due to the loss of antioxidant molecules or other factors important for redox homeostasis from its membranes, which could lead to rapid propagation of Golgi-derived lipid peroxides. For example, human UbiA-domain containing protein 1 (UBIAD1) is a Golgi-localized prenyltransferase that produces the antioxidant CoQ10 (also known as Coenzyme Q10 or ubiquinone), which protects cells from oxidative stress^64^. Golgi stress-induced redistribution of UBIAD1 or other factors such as Parkin, a *trans*-Golgi network-localized E3 ubiquitin ligase that is protective against ER and oxidative stress^65,66^, or the glutaredoxins Grx6/Grx7^67^ to other compartments might thus be related to lipid peroxide formation.

Ferroptosis has emerged as a regulated necrotic cell death pathway^6^, which is explored in multiple disease contexts including periventricular leukomalacia, acute kidney failure/nephrotic tubular death, cancer and neurodegeneration^7,68^. Therefore, ferroptosis modulators are clinically of great interest and have substantial therapeutic potential. For instance, ferroptosis induction is actively pursued as novel treatment strategy to eradicate cancer cells^69–72^ and several FDA-approved drugs including sorafenib, sulfasalazine, tolperisone (lanperisone) and artesunate were shown to induce ferroptosis^41,73,74^ suggesting that their beneficial effects to treat malignant cells could be related to this cell death process. In light of our findings that ferroptosis inducers used at low doses are able to reduce rather than increase BFA-induced lipid peroxidation and to partially restore Golgi homeostasis, a cautionary note on the selection of sufficiently high drug doses that do not stimulate a redox adaptation response with potentially unwanted protumorigenic consequences to treat patients seems to be warranted.

## Methods

### Reagents

Indicated chemicals were obtained from companies (in parentheses): brefeldin A (Sigma-Aldrich), golgicide A (Sigma-Aldrich), AG1478/tyrphostin (Sigma-Aldrich), Erastin (MedChem Express), ferrostatin-1 (Sigma-Aldrich), liproxstatin-1 (Sigma-Aldrich), NOX1/4 inhibitor (GKT137831, Cayman Chemical), Sulfasalazine (Sigma-Aldrich), Sorafenib (Sigma-Aldrich), RSL3 (MedChem Express), Glutathione (Sigma-Aldrich), *N*-acetyl-cysteine (Sigma-Aldrich), Ciclopirox olamine (CPX, Sigma-Aldrich), LOXi (PD-146176, Santa Cruz Biotechnology), Trolox (Sigma-Aldrich), Prankulast (Biomol), tunicamycin (Sigma-Aldrich), erastin (MedChemExpress), BSO (Santa Cruz Biotechnology), cisplatin (Santa Cruz Biotechnology), doxorubicin (Sigma-Aldrich), 2-ME (Sigma-Aldrich), PPG (Sigma-Aldrich),

Nutlin-3 (Sigma-Aldrich), vildagliptin (Sigma-Aldrich), Q-VD-OPh hydrate (Sigma-Aldrich), bafilomycin A (Sigma-Aldrich), nocodazole (Sigma-Aldrich), panobinostat (Sigma-Aldrich), doxorubicin (Sigma-Aldrich). AMF-26 was synthesized by Merck KGaA^23^.

### Cell culture

HeLa, A549, Panc-1, HT-29, DU145, primary lung fibroblasts were grown at 37° C in 5% CO_2_ in DMEM High-Glucose media (Life Technologies) supplemented with 10% heat-inactivated FBS (Life Technologies) and 1% penicillin/streptomycin. Jurkat cells were grown in RPMI 1640 medium (Life Technologies) supplemented with 10% heat-inactivated FBS (Life Technologies) and 1% penicillin/streptomycin. All cell lines with the exception of primary lung fibroblasts were obtained from American Type Culture Collection (ATCC). Primary human pulmonary fibroblasts were obtained from Epithelix. For low cystine conditions cystine-free medium was purchased (Life Technologies) supplemented with 10% dialyzed FBS and mixed with standard DMEM to reach the desired cystine concentration.

### Cell viability and colony formation assays

The impact of various Golgi stress-inducing compounds on cell viability was analyzed using the CellTiter-Blue (CTB) assay (Promega). To determine changes in cell viability, 3000 cells were seeded in full medium in 96-well plates (Greiner Bio One) 24 h prior to the treatment. Cells were then treated for 72 h with the indicated concentration of compounds. Cell viability was analyzed using the CTB assay following the manufacturer’s instructions. For in vitro clonogenic assays, 200 cells were seeded in six-well plate culture dishes 24 h prior to compound treatment. Cells were then treated for 14 days with the mentioned concentration of compounds, and the number of colonies was counted after staining with 1 mL 0.01% (w/v) crystal violet.

### Determination of intracellular ROS levels

Twenty-four hours prior to the treatment, 10^5^ HeLa cells were seeded in six-well plates. Cells were treated with the indicated compounds for the duration specified in the text. Before ROS analysis, the cell culture medium was removed and dihydroethidium (DHE, Sigma-Aldrich) diluted in 500 μl Hanks Balanced Salt Solution (HBSS, Gibco) was added at a final concentration of 5 μM to each well. Cells were then stained for 15 min at room temperature before they were gently harvested using Trypsin-EDTA Solution (Gibco), and ROS levels immediately analyzed using a BD FACS Aria™ III cell sorter (Fig. 1a and Supplementary Fig. 1a). For the analysis of lipid peroxide formation, 10^4^ HeLa cells were seeded in 96-well plates (black). Twenty-four hours later cells were treated with the indicated compounds using DMEM supplemented with 5 µM Liperfluo reagent (DOJINDO) and immediately placed into the IncuCyte® live cell analysis system (Essen BioScience). Depending on the experiment, images were taken every 0.5, 1, or 2 h over the indicated treatment duration. Data are presented as average green fluorescence intensity over time. Signal intensity per well was derived from the average of four pictures taken per well, and for each condition and genotype at least three independent wells were analyzed. Average green fluorescence intensity was analyzed using IncuCyte Software 2016b. Green confluency mask was defined with a lower and upper threshold to exclude background and apoptotic cells, which present a higher fluorescence.

### Measurement of cellular GSH content

To measure the intracellular glutathione levels, 10^5^ HeLa were seeded in six-well plate and treated with the indicated compounds for the duration specified in the text. Cell were then trypsinized and glutathione level were analyzed as previously described^75^.

### Immunofluorescence, image acquisition and data analysis

1500 cells per well were seeded in 80 µl medium in 96-wells plates with clear flat bottoms for imaging (Greiner) and treated in fresh medium 24 h later. After incubation with indicated concentrations of drugs or vehicle (DMSO) for 72 h, cells were fixed with 4% paraformaldehyde (Electron Microscopy [USA]) for 20 min at room temperature, washed once with PBS and permeabilized with 0.05% Triton X-100 (Amresco) in PBS for 20 min. After washing, primary antibodies against GM130 (1:100, sc-16268 Santa-Cruz) diluted in 5% normal donkey serum (Jackson ImmunoResearch) were added for overnight incubation at 4° C. The next day cells were washed three times with PBS and incubated with appropriate secondary antibody (1:2000, Life Technologies) and Hoechst (1:2500, Life Technologies) diluted in 5% normal donkey serum for one hour. Finally, samples were washed five times with PBS. Pictures were acquired with an Olympus Biosystems IX81 inverted microscope at 20 × magnification using Olympus ScanR 2.5.0. acquisition software. For each replicate 9 fields per well were acquired.

### Image processing and feature extraction

Image data analysis was performed using the software KNIME^76^. Segmentation and feature extraction was done as described before^77,78^. In brief, nuclei were segmented using an Otsu thresholding method^79^. Then nuclei were extended to the border of the cell body using a Voronoi algorithm set to stop when the intensity dropped below a defined threshold. The vesicles composing the Golgi apparatus were identified with an Ostu global thresholding method^79^ combined with a Bersen local thresholding method^80^. The structures identified this way were then mapped back to their corresponding cell using the pre-defined cellular masks.

### Protein extraction and western blotting

Whole cell lysates were prepared from 2 × 10^5^ cells treated with the indicated compounds for the duration specified in the text. After washing with ice-cold PBS, cells were lysed using RIPA buffer containing protease and phosphatase inhibitors (Roche Applied Science). Sonicated cell lysates were then centrifuged at 4° C for 15 min. In general, 50 μg protein lysate was loaded per lane and separated on 4–12% gradient SDS-PAGE (Thermo Scientific) gels and transferred to FL-PVDF membranes (Immobilon). Primary antibody incubation was performed overnight at 4° C. Proteins were visualized using far-infrared dye conjugated with secondary antibodies (Li-COR) on a LI-COR Odyssey scanner using Image Studio software (LI-COR). Following antibodies and dilutions were used for immunoblotting: GRP78 (#3177, Cell Signaling Technology [CST]), 1:1000), p-eIF2α (#3398, CST, 1:1000), GSR (#ab16801, Abcam, 1:1000), xCT/SLC7A11 (#12691, CST, 1:1000), cleaved-PARP (#5625, CST, 1:1000), ACSL4 (sc-271800, Santa Cruz Biotechnology, 1:500), GPX4 (#ab41787, Abcam, 1:1000), NRF2 (#12721, CST, 1:1000), NOX1 (#ab121009, Abcam, 1:1000), β-actin (#3700, CST, 1:5000), SLC7A5/LAT1 (#5347, CST, 1:1000), TXNRD1 (#11117, Proteintech, 1:1000), p-H2A.X(Ser139) (#9718, CST, 1:1000), DYKDDDDK Tag Antibody (anti-FLAG) (#2368, CST, 1:1000)

### Generation of stable knockdown or overexpression cell lines

To generate lentivirus for knockdown or overexpression studies 8 × 10^5^ HEKT293 cells were seeded in 6 cm dishes. 24 h afterwards the cells were transfected with plasmids encoding ΔVPR and pCG (VSV-G envelope protein expression vector) in addition to 1 μg of shRNA or overexpression vector using LT1 reagent transfection (Mirus) at a ratio of 3:1. 12 h post-transfection, media was aspirated and replaced with GlutaMAX (Invitrogen) media plus 30 % IFS + 5 mM L-glutamine. Lentiviral supernatants were collected 48 h post transfection of plasmids, and virus-containing medium was filtered (0.45 μm) to remove cellular debris. For lentiviral infection, cells were plated at a density of 1.5 × 10^5^ in 6 cm dishes 24 h prior to lentiviral transduction. For generation of stable overexpression cell lines, 3 mL of viral supernatant was added to the cells after media aspiration, and polybrene (Sigma Aldrich) was added to a final concentration of 8 μg/ml. For generation of knockdown cell lines, the culture medium was replaced by 3 ml of standard cell culture media supplemented with 8 μg/ml polybrene before addition of lentiviral particles carrying shRNA constructs. 200 μL (A549) or 400 μL (HeLa, Panc-1) of viral supernatant was added to the media. 24 h later, viral supernatant was replaced with media supplemented with 2 μg/ml Puromycin (VWR) to select for infected cells. Details of cDNA and shRNA sources and primer sequences are given in Tables 1–4.

### Gaussia luciferase (Gluc) protein secretion assay

HeLa stably overexpressing Gluc-flag were seeded at a density of 5000 cells per well in 96-well assay plates. 24 h later the ferroptosis-modulating compounds (erastin, sorafenib, ferrostatin-1, GSH) were added in fresh medium and incubated for 24 h. Thereafter, medium was aspirated and replaced with medium containing BFA plus the respective ferroptosis-modulating compound, and the cells were grown for additional 2 h before the medium was exchanged with normal DMEM (without addition of any chemicals). 15 min after this medium exchange 50 µl of culture supernatant was transferred to a white, opaque 96-well plate. A volume of 20 µl freshly prepared Gaussia luciferase (Gluc) flash assay reagent (Pierce) was added and the luminescent signal was read after a 10 s integration time on a GloMax®-Multi reader (Promega).

## Electronic supplementary material

Supplementary information

## Data Availability

The authors declare that all data supporting the findings of this study are available within the paper and its supplementary information files.

## References

[1] Arya R, White K (2015). Cell death in development: signaling pathways and core mechanisms. Semin. Cell Dev. Biol..

[2] Shimada K (2016). Global survey of cell death mechanisms reveals metabolic regulation of ferroptosis. Nat. Chem. Biol..

[3] Dong T, Liao D, Liu X, Lei X (2015). Using small molecules to dissect non-apoptotic programmed cell death: Necroptosis, ferroptosis, and pyroptosis. Chembiochem.

[4] Conrad M, Angeli JPF, Vandenabeele P, Stockwell BR (2016). Regulated necrosis: disease relevance and therapeutic opportunities. Nat. Rev. Drug. Discov..

[5] Laster SM, Wood JG, Gooding LR (1988). Tumor necrosis factor can induce both apoptotic and necrotic forms of cell lysis. J. Immunol..

[6] Dixon SJ (2012). Ferroptosis: an iron-dependent form of nonapoptotic cell death. Cell.

[7] Xie Y (2016). Ferroptosis: process and function. Cell Death Differ..

[8] Stipanuk MH, Dominy JE, Lee JI, Coloso RM (2006). Mammalian cysteine metabolism: new insights into regulation of cysteine metabolism. J. Nutr..

[9] McBean GJ (2012). The transsulfuration pathway: a source of cysteine for glutathione in astrocytes. Amino Acids.

[10] Belalcázar AD, Ball JG, Frost LM, Valentovic MA, Wilkinson J (2013). Transsulfuration is a significant source of sulfur for glutathione production in human mammary epithelial cells. ISRN Biochem..

[11] Garg SK, Yan Z, Vitvitsky V, Banerjee R (2011). Differential dependence on cysteine from transsulfuration versus transport during T cell activation. Antioxid. Redox Signal..

[12] Imai H, Nakagawa Y (2003). Biological significance of phospholipid hydroperoxide glutathione peroxidase (PHGPx, GPx4) in mammalian cells. Free Radic. Biol. Med..

[13] Doll S (2016). ACSL4 dictates ferroptosis sensitivity by shaping cellular lipid composition. Nat. Chem. Biol..

[14] Yuan H, Li X, Zhang X, Kang R, Tang D (2016). Identification of ACSL4 as a biomarker and contributor of ferroptosis. Biochem. Biophys. Res. Commun..

[15] Lee SA, Kim YJ, Lee CS (2013). Brefeldin a induces apoptosis by activating the mitochondrial and death receptor pathways and inhibits focal adhesion kinase-mediated cell invasion. Basic. Clin. Pharmacol. Toxicol..

[16] Shao RG, Shimizu T, Pommier Y (1996). Brefeldin A is a potent inducer of apoptosis in human cancer cells independently of p53. Exp. Cell Res..

[17] Pommepuy I (2003). Brefeldin A induces apoptosis and cell cycle blockade in glioblastoma cell lines. Oncology.

[18] Wlodkowic D, Skommer J, Pelkonen J (2007). Brefeldin A triggers apoptosis associated with mitochondrial breach and enhances HA14-1- and anti-Fas-mediated cell killing in follicular lymphoma cells. Leuk. Res..

[19] Guo H, Tittle TV, Allen H, Maziarz RT (1998). Brefeldin A-mediated apoptosis requires the activation of caspases and is inhibited by Bcl-2. Exp. Cell Res..

[20] Sáenz JB (2009). Golgicide a reveals essential roles for GBF1 in Golgi assembly and function. Nat. Chem. Biol..

[21] Ohashi Y (2012). AMF-26, a novel inhibitor of the Golgi system, targeting ADP-ribosylation factor 1 (Arf1) with potential for cancer therapy. J. Biol. Chem..

[22] Zeghouf M, Guibert B, Zeeh JC, Cherfils J (2005). Arf, Sec7 and Brefeldin A: a model towards the therapeutic inhibition of guanine nucleotide-exchange factors. Biochem. Soc. Trans..

[23] Ignashkova TI (2017). Cell survival and protein secretion associated with Golgi integrity in response to Golgi stress-inducing agents. Traffic.

[24] Ramírez-Peinado S (2017). TRAPPC13 modulates autophagy and the response to Golgi stress. J. Cell. Sci..

[25] Mishev K, Dejonghe W, Russinova E (2013). Small molecules for dissecting endomembrane trafficking: a cross-systems view. Chem. Biol..

[26] Lu ShellyC, Glutathione MD (2014). Synthesis. Biochim. Biophys. Acta.

[27] Yang WS (2014). Regulation of ferroptotic cancer cell death by GPX4. Cell.

[28] Yamanaka K (2012). A novel fluorescent probe with high sensitivity and selective detection of lipid hydroperoxides in cells. RSC Adv..

[29] Reiling JH (2013). A CREB3-ARF4 signalling pathway mediates the response to Golgi stress and susceptibility to pathogens. Nat. Cell Biol..

[30] Zhang B (2018). Arf1 regulates the ER–mitochondria encounter structure (ERMES) in a reactive oxygen species-dependent manner. FEBS J..

[31] Friedmann Angeli JP (2014). Inactivation of the ferroptosis regulator Gpx4 triggers acute renal failure in mice. Nat. Cell Biol..

[32] Zilka O (2017). On the mechanism of cytoprotection by Ferrostatin-1 and Liproxstatin-1 and the role of lipid peroxidation in ferroptotic cell death. ACS Cent. Sci..

[33] Pan H (2008). A novel small molecule regulator of guanine nucleotide exchange activity of the ADP-ribosylation factor and Golgi membrane trafficking. J. Biol. Chem..

[34] Kagan VE (2016). Oxidized arachidonic and adrenic PEs navigate cells to ferroptosis. Nat. Chem. Biol..

[35] Kuhn H, Banthiya S, Van Leyen K (2015). Mammalian lipoxygenases and their biological relevance. Biochim. Biophys. Acta Mol. Cell Biol. Lipids.

[36] Dvash, E., Har-Tal, M., Barak, S., Meir, O. & Rubinstein, M. Leukotriene C 4 is the major trigger of stress-induced oxidative DNA damage. *Nat. Commun*. **6**, 10112 (2015). 10.1038/ncomms10112 PMC4682057 26656251

[37] Xie Y (2017). The tumor suppressor p53 limits ferroptosis by blocking DPP4 activity. Cell Rep..

[38] Tarangelo A (2018). p53 suppresses metabolic stress-induced ferroptosis in cancer cells. Cell Rep..

[39] Jennis M (2016). An African-specific polymorphism in the TP53 gene impairs p53 tumor suppressor function in a mouse model. Genes Dev..

[40] Jiang L (2015). Ferroptosis as a p53-mediated activity during tumour suppression. Nature.

[41] Dixon SJ (2014). Pharmacological inhibition of cystine-glutamate exchange induces endoplasmic reticulum stress and ferroptosis. eLife.

[42] Dixon SJ (2015). Human haploid cell genetics reveals roles for lipid metabolism genes in nonapoptotic cell death. ACS Chem. Biol..

[43] Lu J, Holmgren A (2014). The thioredoxin antioxidant system. Free Radic. Biol. Med..

[44] Mandal PK (2010). System xc- and thioredoxin reductase 1 cooperatively rescue glutathione deficiency. J. Biol. Chem..

[45] Harris IS (2015). Glutathione and thioredoxin antioxidant pathways synergize to drive cancer initiation and progression. Cancer Cell.

[46] Sun X (2016). Activation of the p62-Keap1-NRF2 pathway protects against ferroptosis in hepatocellular carcinoma cells. Hepatology.

[47] Zhu Shan, Zhang Qiuhong, Sun Xiaofan, Zeh Herbert J., Lotze Michael T., Kang Rui, Tang Daolin (2017). HSPA5 Regulates Ferroptotic Cell Death in Cancer Cells. Cancer Research.

[48] Yu X, Long YC (2016). Crosstalk between cystine and glutathione is critical for the regulation of amino acid signaling pathways and ferroptosis. Sci. Rep..

[49] Ishii T, Bannai S (1981). Mechanism of growth stimulation of L1210 cells by 2-mercaptoethanol in vitro. J. Biol. Chem..

[50] Hayano M, Yang WS, Corn CK, Pagano NC, Stockwell BR (2016). Loss of cysteinyl-tRNA synthetase (CARS) induces the transsulfuration pathway and inhibits ferroptosis induced by cystine deprivation. Cell Death Differ..

[51] van Raam BJ, Lacina T, Lindemann RK, Reiling JH (2017). Secretory stressors induce intracellular death receptor accumulation to control apoptosis. Cell Death Dis..

[52] Chen ZH, Yoshida Y, Saito Y, Noguchi N, Niki E (2006). Adaptive response induced by lipid peroxidation products in cell cultures. FEBS Lett..

[53] Uchida K (2003). 4-Hydroxy-2-nonenal: a product and mediator of oxidative stress. Prog. Lipid Res..

[54] Gao M (2016). Ferroptosis is an autophagic cell death process. Cell Res..

[55] Marinari UM (1987). Inhibition of liver Golgi glycosylation activities by carbonyl products of lipid. Free. Radic. Res..

[56] Rafols JA (1995). Global brain ischemia and reperfusion: Golgi apparatus ultrastructure in neurons selectively vulnerable to death. Acta Neuropathol..

[57] Guiney SJ, Adlard PA, Bush AI, Finkelstein DI, Ayton S (2017). Ferroptosis and cell death mechanisms in Parkinson’s disease. Neurochem. Int..

[58] Gaschler MM, Stockwell BR (2017). Lipid peroxidation in cell death. Biochem. Biophys. Res. Commun..

[59] Jiang Z (2011). The role of the Golgi apparatus in oxidative stress: is this organelle less significant than mitochondria?. Free Radic. Biol. Med..

[60] Farber-Katz SE (2014). DNA damage triggers golgi dispersal via DNA-PK and GOLPH3. Cell.

[61] Gendarme M (2017). Image-based drug screen identifies HDAC inhibitors as novel Golgi disruptors synergizing with JQ1. Mol. Biol. Cell.

[62] Dinter A, Berger EG (1998). Golgi-disturbing agents. Histochem. Cell. Biol..

[63] Hedrick E, Crose L, Linardic CM, Safe S (2015). Histone deacetylase inhibitors inhibit rhabdomyosarcoma by reactive oxygen species-dependent targeting of specificity protein transcription factors. Mol. Cancer Ther..

[64] Mugoni V (2013). Ubiad1 is an antioxidant enzyme that regulates eNOS activity by CoQ10 synthesis. Cell.

[65] Ledesma MD, Galvan C, Hellias B, Dotti C, Jensen PH (2002). Astrocytic but not neuronal increased expression and redistribution of parkin during unfolded protein stress. J. Neurochem..

[66] Kubo SI (2001). Parkin is associated with cellular vesicles. J. Neurochem..

[67] Mesecke N, Spang A, Deponte M, Herrmann JM (2008). A novel group of glutaredoxins in the cis-Golgi critical for oxidative stress resistance. Mol. Biol. Cell..

[68] Yang WS, Stockwell BR (2016). Ferroptosis: death by lipid peroxidation. Trends Cell Biol..

[69] Kim SE (2016). Ultrasmall nanoparticles induce ferroptosis in nutrient-deprived cancer cells and suppress tumour growth. Nat. Nanotechnol..

[70] Chen L (2015). Erastin sensitizes Glioblastoma cells to temozolomide by restraining xCT and cystathionine-gamma-lyase function. Oncol. Rep..

[71] Cramer SL (2016). Systemic depletion of L-cyst(e)ine with cyst(e)inase increases reactive oxygen species and suppresses tumor growth. Nat. Med..

[72] Viswanathan VS (2017). Dependency of a therapy-resistant state of cancer cells on a lipid peroxidase pathway. Nature.

[73] Shaw AT (2011). Selective killing of K-ras mutant cancer cells by small molecule inducers of oxidative stress. Proc. Natl. Acad. Sci. U. S. A..

[74] Eling N, Reuter L, Hazin J, Hamacher-Brady A, Brady NR (2015). Identification of artesunate as a specific activator of ferroptosis in pancreatic cancer cells. Oncoscience.

[75] Rahman I, Kode A, Biswas S (2006). Assay for quantitative determination of glutathione and glutathione disulfide levels using enzymatic recycling method. Nat. Protoc..

[76] Dietz, C. & Berthold, M. R. in *Focus on**Bio-Image Informatics* (eds. De Vos, W. H., Munck, S. & Timmermans, J.-P.) 179–197 (Springer International Publishing, Switzerland, 2016).

[77] Carpenter AE (2006). CellProfiler: image analysis software for identifying and quantifying cell phenotypes. Genome Biol..

[78] Held M (2010). CellCognition: time-resolved phenotype annotation in high-throughput live cell imaging. Nat. Methods.

[79] Otsu N (1979). A threshold selection method from gray-level histograms. IEEE Trans. Syst. Man. Cybern..

[80] Chen S, Leung H (2004). Chaotic spread spectrum watermarking for remote sensing images. J. Electron. Imaging.

[81] Reiling JH (2011). A haploid genetic screen identifies the major facilitator domain containing 2A (MFSD2A) transporter as a key mediator in the response to tunicamycin. Proc. Natl Acad. Sci. USA.

