# Supplementary material for: Golgi stress mediates redox imbalance and ferroptosis in human cells
Source: Commun Biol. 2018 Nov 28;1:210. doi: 10.1038/s42003-018-0212-6 (PMC6262011; doi:10.1038/s42003-018-0212-6)

SUPPLEMENTARY FIGURE 1

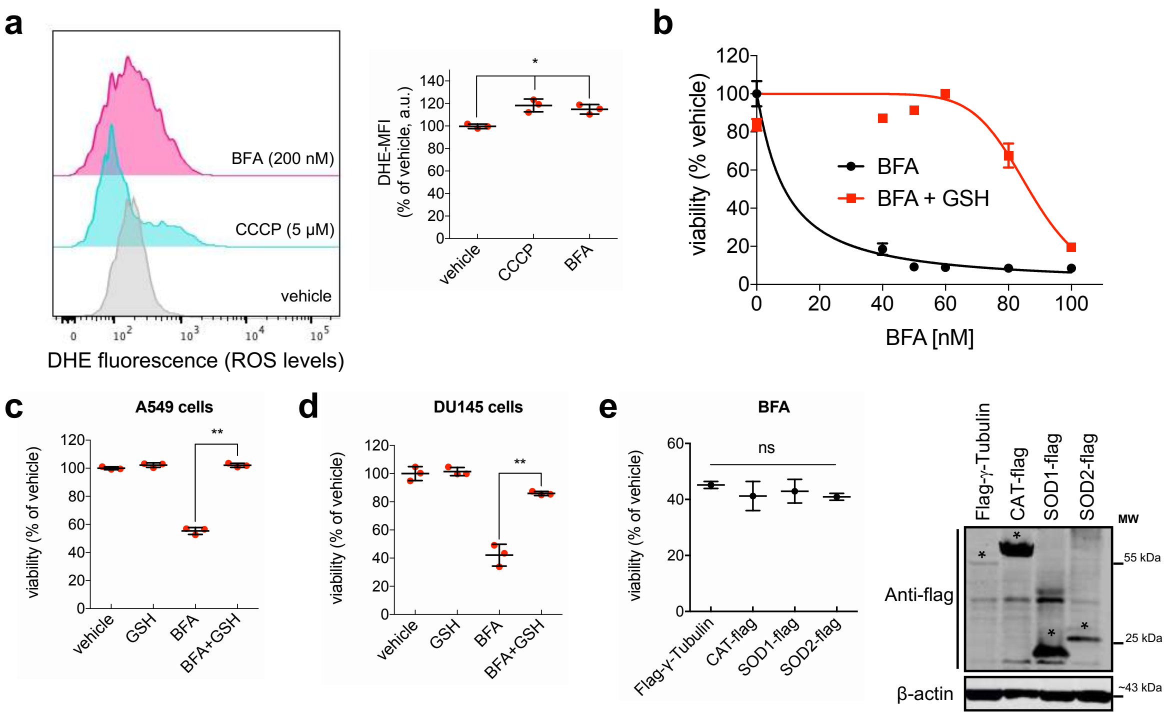

Supplementary Figure 1: Impact of GSH and several ROS-degrading enzymes on viability in response to BFA treatment

(a) Analysis of intracellular reactive oxygen species (ROS) accumulation using dihydroethidium (DHE) staining and flow cytometry of Jurkat cells treated for 48 h treatment with either 200 nM BFA or 5 µM CCCP as a positive control (left graph). Right graph shows quantification of the mean fluorescence intensities (MFI) of samples shown in the left histogram, $*P < 0.05$; a.u.: arbitrary unit. (b) Relative viability of HeLa cells treated for 72 h with increasing concentrations of BFA in the presence or absence of 2 mM glutathione (GSH). (c and d) Relative viability of A549 (c) and DU145 (d) cells after treatment with BFA (50 nM BFA for A549 cells, 65 nM BFA for DU145 cells) for 72 h in the presence or absence of 2 mM glutathione (GSH), $**P < 0.01$. (e) HeLa cells were stably transduced with the indicated constructs, and relative survival was assessed following treatment with 35 nM BFA for 72 h. Expression levels of γ-Tubulin, CAT, SOD1, and SOD2 are shown by western blot (right panel). Asterisk symbols denote bands of overexpressed proteins of interest. (c-d) Cell survival was determined by the CTB assay. (a, c-e): Statistical analyses were performed using Student's two-tailed t-test. Shown are representative examples of three independent experiments, and three wells per treatment condition were measured.

Center bars indicate the mean, error bars indicate the SD. Scanned images of unprocessed blots are shown in Supplementary Figure 7.

SUPPLEMENTARY FIGURE 2

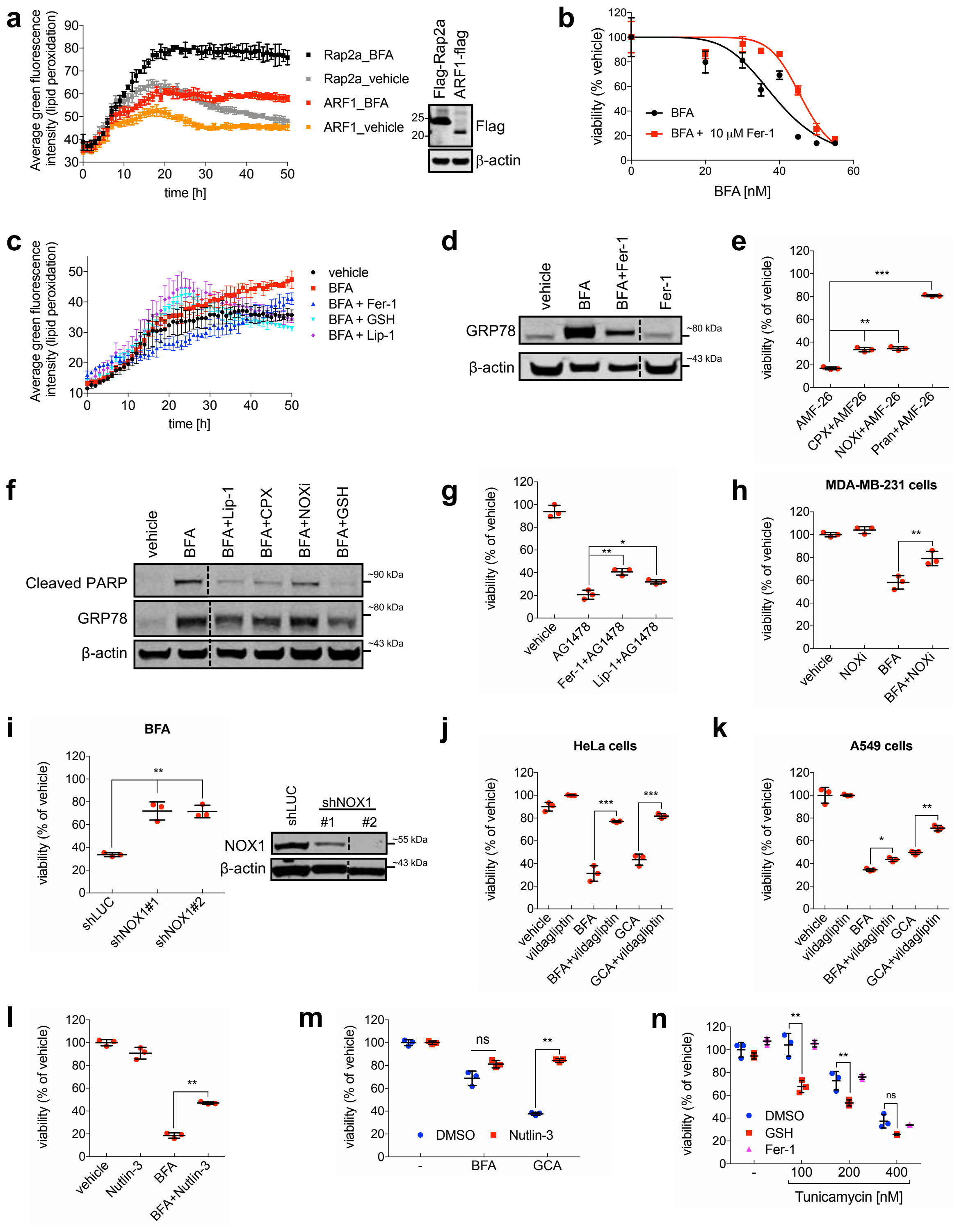

Supplementary Figure 2: Inhibition of ferroptosis protects from Golgi stress

(a) HeLa cells stably overexpressing ARF1-flag (WT) or Flag-Rap2a (control) were left untreated or treated with 40 nM BFA for the indicated time, and lipid peroxidation was measured over the time of treatment using Liperfluo staining. Statistical significance between the different treatment conditions and genotypes was calculated using a two-way ANOVA test, and the average fluorescence intensity for each condition and genotype was derived from four pictures taken from four wells. BFA-treated ARF1-flag overexpression cells had significantly reduced lipid peroxide accumulation compared to control (Flag-Rap2a) overexpression cells starting at nine hours of treatment; $P < 0.001$. Western blot shows expression levels of Flag-Rap2a and ARF1-flag, β-actin serves as loading control. (b) Dose-response curves of HeLa cells treated for 72 h with 20 nM, 30 nM, 35 nM, 40 nM, 45 nM, 50 nM or 55 nM BFA in the presence or absence of 10 μM Fer-1. EC_{50} for BFA: 39 nM, EC_{50} for BFA + Fer-1: 46 nM BFA. Cell viability was assessed using the CTB assay. (c) HeLa cells were treated either with vehicle, 40 nM BFA, or 40 nM BFA in combination with either of three different ferroptosis inhibitors (Fer-1 [10 μM], Lip-1 [1 μM] or GSH [2 mM]). Statistical significance between the different treatment conditions and genotypes was calculated using a two-way ANOVA test, and the average fluorescence intensity for each condition and genotype was derived from four pictures taken from four wells. Co-incubation of BFA with either Fer-1, GSH or Lip-1, respectively, significantly reduced lipid peroxide accumulation compared to BFA-only treatment starting at 15 h (Fer-1), 33 h (GSH) and 38 h (Lip-1) of treatment; $P < 0.001$ (for GSH and Fer-1) and $P<0.05$ (for Lip-1). (d) HeLa cells were treated for 24 h with 40 nM BFA, 15 μM ferrostatin-1 (Fer-1) or a combination thereof before protein extraction and western blot analysis for the indicated proteins. β-actin serves as loading control. Protein lysates were run on the same gel, and dashed lines in blots indicate where irrelevant samples were cropped out. A representative immunoblot of three independent experiments is shown. (e) Relative survival of HeLa cells treated for 72 h with 35 nM AMF-26 in the presence or absence of 1 μM CPX, 4 μM NADPH oxidase1/4 inhibitor (NOXi, GKT137831) or 10 μM pranlukast (Pran), a cysteinyl leukotriene receptor-1 (CysLTR1) inhibitor, $^{**}P < 0.01$; $^{***}P < 0.001$. (f) Lysates of HeLa cells treated for 24 h with the indicated compounds before protein extraction were analyzed by western blot using the antibodies shown. A representative western blot of two independent experiments is shown. Concentrations of compounds

were as follows: 40 nM BFA, 5 μM Lip-1, 1 μM CPX, 4 μM NADPH inhibitor (NOXi/GKT137831) and 2 mM GSH. β-actin was used as a loading control. Protein lysates were run on the same gel, and dashed lines in blots indicate where irrelevant samples were cropped out. (g) Impact of the Golgi-disrupting agent AG1478/tyrphostin in the absence or presence of Fer-1 or Lip-1 on viability of HeLa cells after 72 h of compound treatment. Relative cell viability (survival of compound-treated cells divided by survival of vehicle-treated cells) was measured by the CellTiter-Blue (CTB) assay. Drug concentrations used for this experiment were as follows: AG1478 (20 μM), ferrostatin-1 (Fer-1, 10 μM) or liproxstatin-1 (Lip-1, 5 μM). (h) Relative viability of MDA-MB-231 cells in response to 72 h 40 nM BFA treatment, 4 μM NADPH oxidase1/4 inhibitor (NOXi, GKT137831) or a combination thereof, $^{**}P < 0.01$. (i) Relative viability of HeLa NOX1 knockdown cells after treatment with 30 nM BFA for 72 h, $^{**}P < 0.01$. NOX1 knockdown validation is shown by western blot. Protein lysates were run on the same gel, and dashed lines in blots indicate where irrelevant samples were cropped out. (j) Relative HeLa cell viability as measured by the CellTiter-Blue (CTB) assay (survival of compound-treated cells divided by survival of vehicle-treated cells) after 72 h treatment with 40 nM BFA or 1.5 μM GCA in the presence or absence of 5 μM vildagliptin. $^{***}P < 0.001$. (k) Relative A549 cell viability as measured by the CellTiter-Blue (CTB) assay (survival of compound-treated cells divided by survival of vehicle-treated cells) after 72 h treatment with 70 nM BFA or 2 μM GCA in the presence or absence of 5 μM vildagliptin. $^{*}P < 0.05$, $^{**}P < 0.01$. (l) Relative viability (survival of compound-treated cells divided by survival of vehicle-treated cells) of HeLa cells following 72 h treatment with 40 nM BFA in the presence or absence of 5 μM Nutlin-3. Cell viability was measured using the CellTiter-Blue (CTB) assay. A representative example of three independent experiments is shown, $^{**}P < 0.01$. (m) A549 cells were treated for 72 h with the indicated compounds. Following concentrations were used: 70 nM BFA, 2.5 μM GCA and 5 μM Nutlin-3, $^{**}P < 0.01$. (n) Relative viability of HeLa cells following treatment for 72 h with increasing concentrations of tunicamycin (TM) in the presence or absence of 15 μM ferrostatin-1 (Fer-1) or 2 mM glutathione (GSH), $^{**}P < 0.01$. (e, g-n): Cell survival was determined using the CTB assay; statistical analyses were performed using Student's two-tailed t-test. Center bars indicate the mean, error bars indicate the SD. Shown are representative examples of three independent

experiments, and three wells per treatment condition were measured. Scanned images of unprocessed blots are shown in Supplementary Figure 7.

SUPPLEMENTARY FIGURE 3

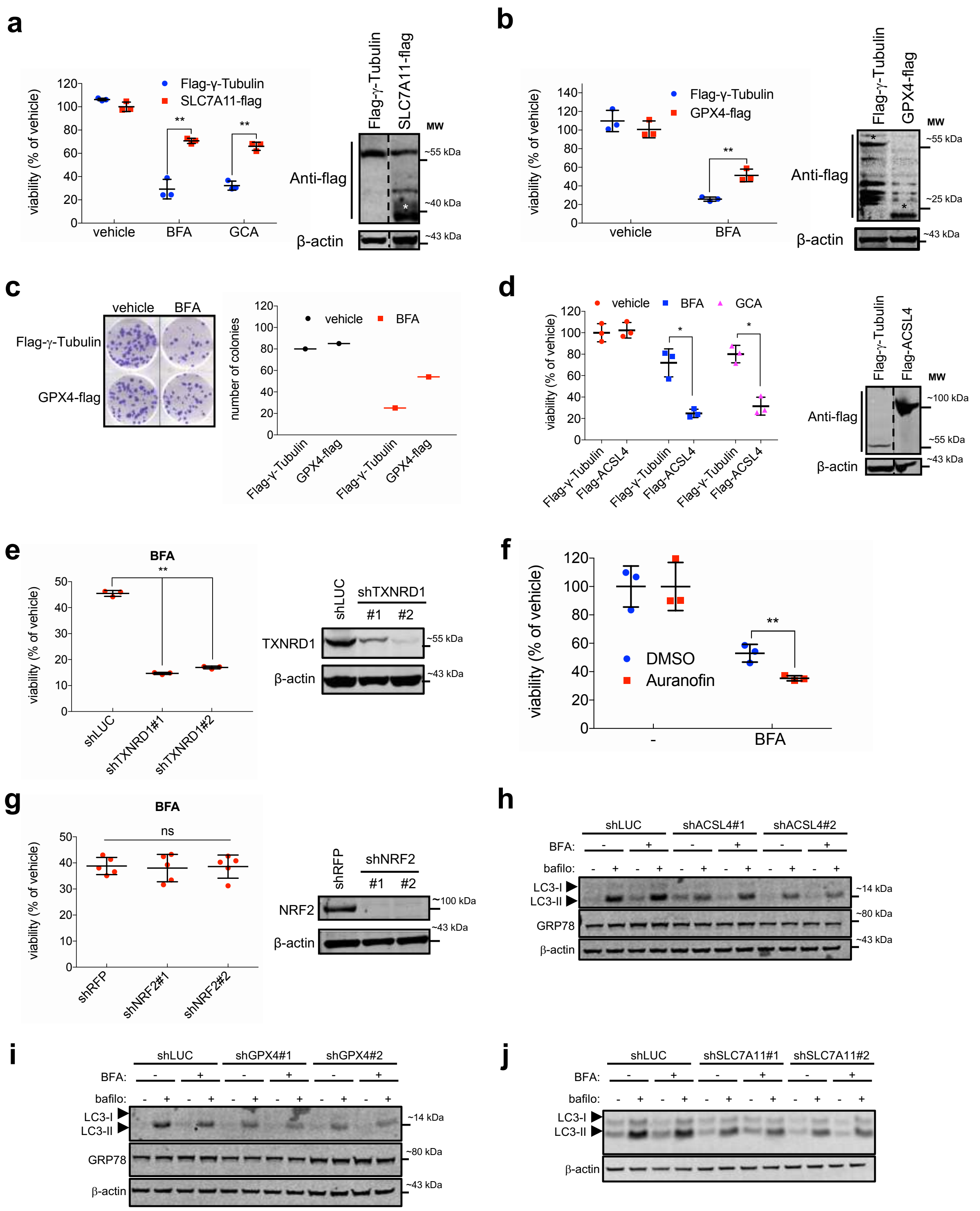

Supplementary Figure 3: Impact of ferroptosis-associated factors on survival and autophagy in response to Golgi stressors.

(a) Relative survival of SLC7A11-flag-overexpressing and overexpression control HeLa cells after treatment with 30 nM BFA or 1.25 µM GCA for 72 h, ** $P < 0.01$. Expression levels of SLC7A11-flag and Flag-γ-Tubulin (control) are shown by western blot. White asterisk represents SLC7A11 band. (b) Relative viability of GPX4-flag and Flag-γ-Tubulin overexpressing HeLa cells after treatment for 72 h with 35 nM BFA, ** $P < 0.01$. Expression levels of GPX4 and γ-Tubulin are shown by western blot. (c) In vitro colony formation assay (n=1) of stable HeLa cells overexpressing epitope-tagged GPX4 or a control protein (γ-Tubulin) treated with 35 nM BFA for 14 days (see also Material and Methods). The graph displays the quantification of the number of colonies formed shown in the left picture. (d) Relative viability of Flag-ACSL4 or Flag-γ-Tubulin (control) overexpressing HeLa cells treated for 72 h with 35 nM BFA and 1.5 µM GCA, * $P < 0.05$. (e) Relative viability of TXNRD1 knockdown and shLUC control HeLa cells after treatment with 40 nM BFA for 72 h, ** $P < 0.01$. (f) Relative viability of HeLa cells treated for 72 h with 30 nM BFA in the presence or absence of 0.75 µM auranofin (TXNRD1 inhibitor), ** $P < 0.01$. (g) Relative viability of NRF2 knockdown HeLa cells following treatment with 35 nM BFA for 72 h. Expression levels of NRF2 in NRF2-depleted and control (shRFP) HeLa cells are shown by western blot analysis (right panel). (a, b, d-g) Cell survival was determined using the CTB assay. Statistical analyses were performed using Student's two-tailed t-test. Shown are representative examples of three independent experiments, and three (a, b, d-f) or five (g) wells per treatment condition were measured. Center bars indicate the mean, error bars indicate the SD. (h-j) HeLa control and ACSL4 (h), GPX4 (i) and SLC7A11 (j) knockdown cells were vehicle-treated or treated with 40 nM BFA for 48 h either in the presence (+) or absence (-) of 50 nM bafilomycin A for the last three hours of the experiment before cell lysis and immunoblot analysis using the indicated antibodies. Representative western blots of three independent experiments are shown. Scanned images of unprocessed blots are shown in Supplementary Figure 7.

SUPPLEMENTARY FIGURE 4

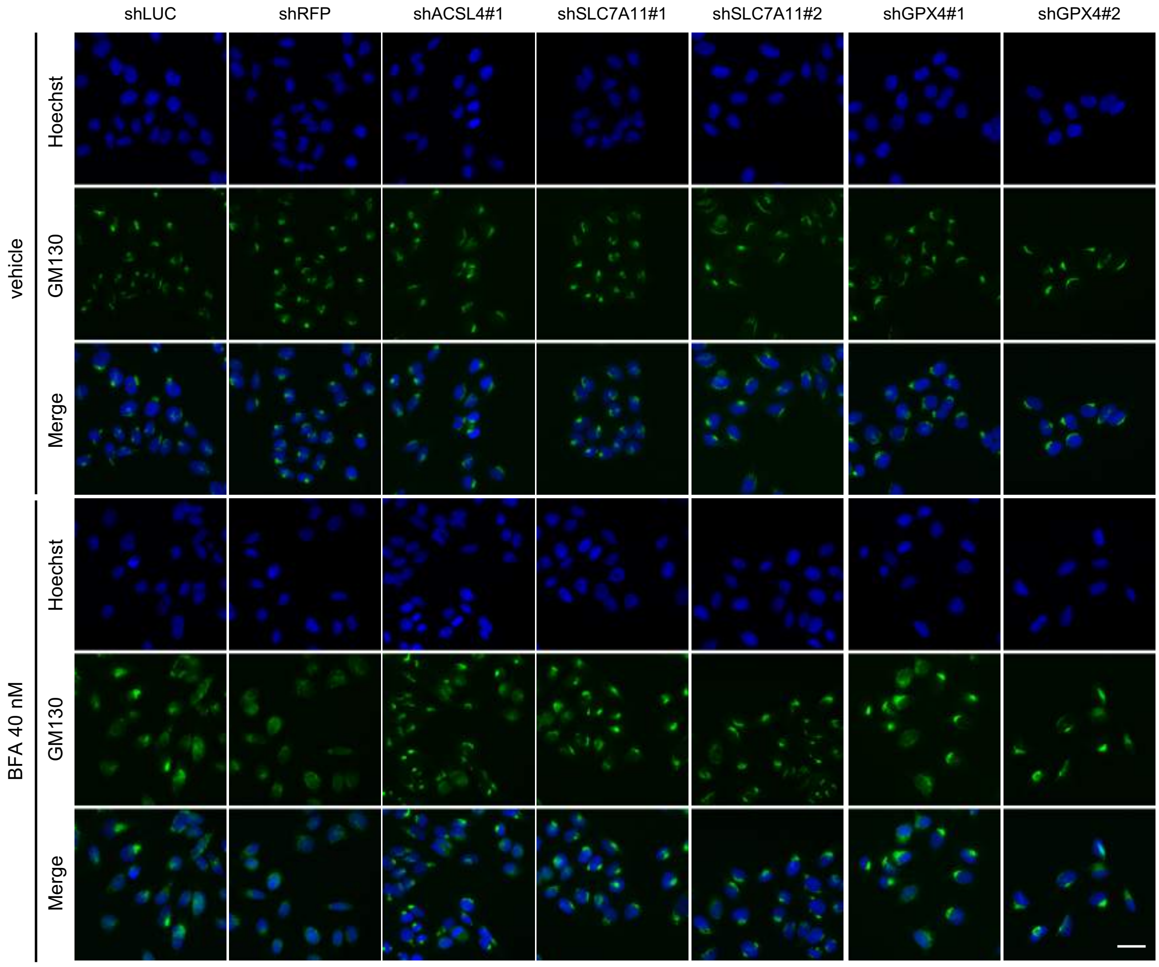

Supplementary Figure 4: ACSL4, SLC7A11 and GPX4 downregulation protects from BFA-induced Golgi dispersal

HeLa cells stably transduced with control hairpins (shLUC or shRFP) or hairpins against ACSL4, SLC7A11 and GPX4 were vehicle-treated or treated with 40 nM BFA for 72 h before fixation and immunofluorescence staining for GM130 to assess Golgi dispersal. For quantification of Golgi areas of all genotypes shown, see Figure 4f. Scale bar: 50 µm.

SUPPLEMENTARY FIGURE 5

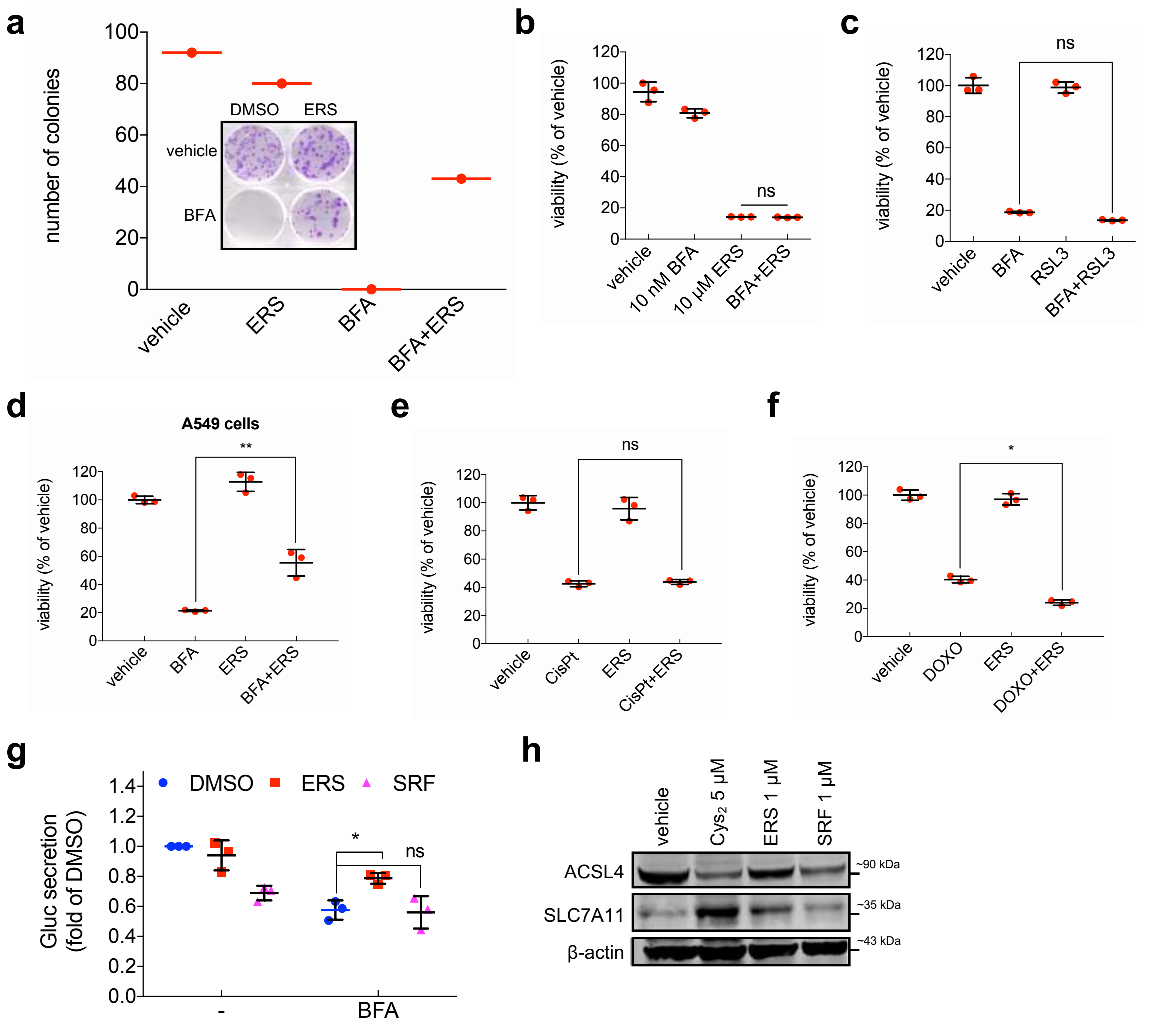

Supplementary Figure 5: Effect of low-dose erastin treatment in combination with BFA or other stressors on survival and protein secretion

(a) HeLa cells were treated with 35 nM BFA for 14 days in the presence or absence of 1 µM erastin (ERS), and the effect on colony formation was assessed. The quantification of colonies was derived from the inset picture (one experiment was performed). (b) Relative HeLa cell viability (survival of compound-treated cells divided by survival of vehicle-treated cells) after 72 h treatment with a low dose of BFA (10 nM) and a high dose of erastin (10 µM). One representative example of three independent experiments is shown, each time measuring three wells per condition. (c) Relative survival of HeLa cells following treatment with 35 nM BFA for 72 h in the presence or absence of 800 nM RSL3 (GPX4 inhibitor). (d) Relative viability of A549 cells treated with 60 nM BFA for 72 h in the presence or absence of 1 µM erastin (ERS), $^{**}P < 0.01$. (e, f) Relative viability of HeLa cells treated either with 5

μM cisplatin (CisPt) or 65 nM doxorubicin (DOXO) for 72 h in the presence or absence of 1 μM erastin; *$P < 0.05$. (g): *Gaussia* Luciferase secretion assay of HeLa cells stably overexpressing Gluc-flag. Cells were treated with 40 nM BFA alone or in combination with either 1 μM erastin or 1 μM sorafenib for 2 h. Before co-treatment with BFA, cells were grown for 24 h in media containing 1 μM erastin or 1 μM sorafenib. The level of secretory activity of cells was determined as a ratio calculated by dividing the luminescence values of drug-treated samples by the values of the corresponding vehicle control (after background signal subtraction). Plotted are average values based on three wells per genotype and condition (n=1); *$P < 0.05$. (b-g): Statistical analyses were performed using Student's two-tailed t-test. Center bars indicate the mean, error bars indicate the SD. Shown are representative examples of three independent experiments, and three wells per treatment condition were measured. (h) Western blot analysis of HeLa cells treated with 1 μM erastin (ERS), 1 μM sorafenib (SRF), or grown in medium containing a low Cys_2 concentration for 72 h. After SDS-PAGE and protein transfer, membranes were probed with antibodies against ACSL4, SLC7A11 or β-actin (loading control). One representative western blot of three independent experiments is shown. Scanned images of unprocessed blots are shown in Supplementary Figure 7.

SUPPLEMENTARY FIGURE 6

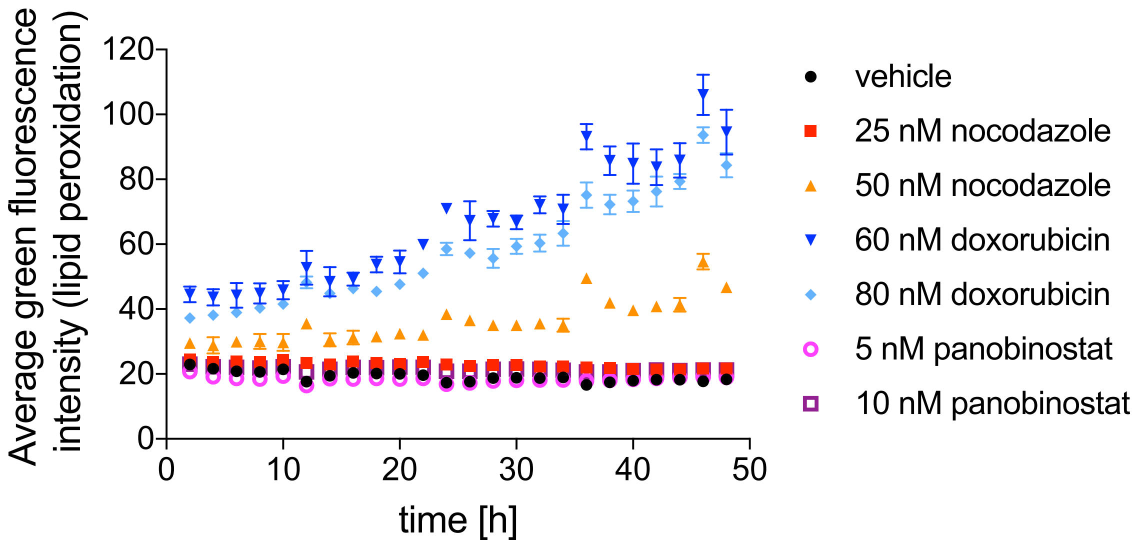

Supplementary Figure 6: Lipid peroxidation in response to Golgi-dispersing compounds

HeLa cells were treated for 48 h with the indicated concentrations of small molecules, and lipid peroxide formation was monitored in real-time using the IncuCyte system in combination with Liperfluo staining. Statistical significance between the different treatment conditions was calculated using a two-way ANOVA test, and the average fluorescence intensity for each condition was derived from four pictures taken per well from four separate wells. 50 nM nocodazole as well as 60 nM or 80 nM doxorubicin treatment caused a significant increase in lipid peroxide production over the entire time course of treatment compared to vehicle; $P < 0.001$. The effect of 5 nM and 10 nM panobinostat on lipid hydroperoxide generation was not significant; 25 nM nocodazole treatment caused no consistent and continuous increase in lipid peroxidation over the entire treatment time, although for some time points there is a very mild increase in lipid peroxidation ($P < 0.05$).

SUPPLEMENTARY FIGURE 7 (Unprocessed blots)

Figure 1e

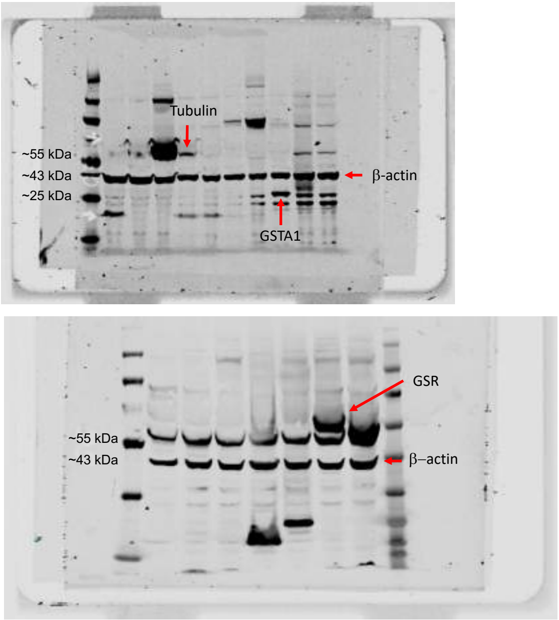

SUPPLEMENTARY FIGURE 7 (Unprocessed blots)

Figure 2d

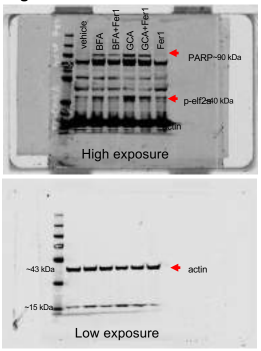

Figure 2i

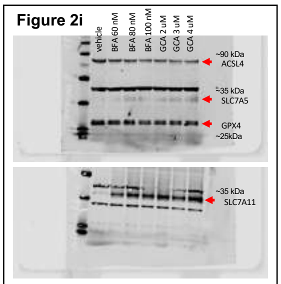

Figure 2j

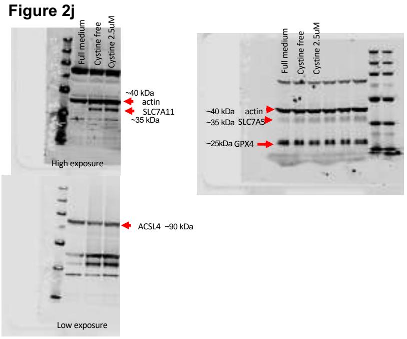

SUPPLEMENTARY FIGURE 7 (Unprocessed blots)

Figure 4a

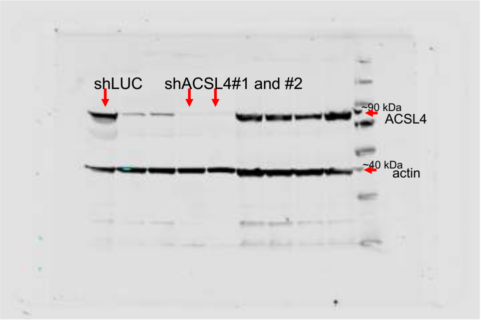

Figure 4b

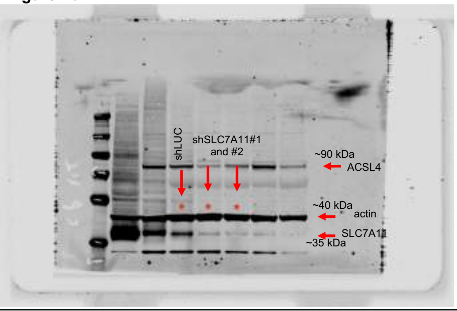

Figure 4c

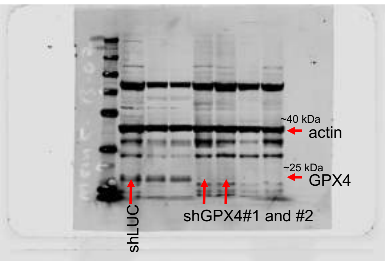

SUPPLEMENTARY FIGURE 7 (Unprocessed blots)

Figure 4d and 4e

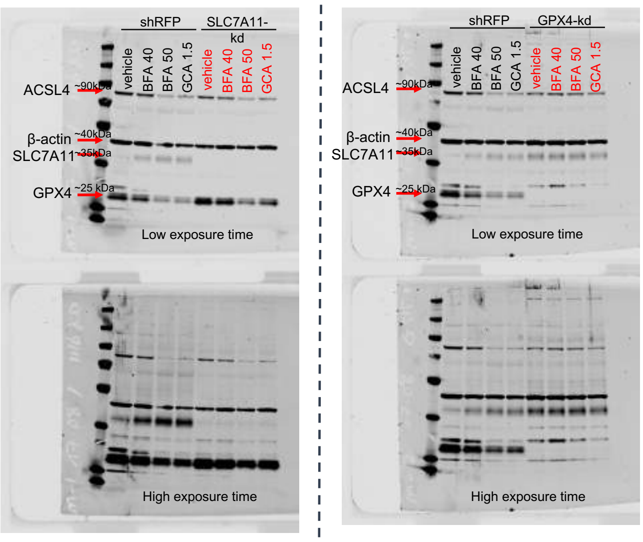

SUPPLEMENTARY FIGURE 7 (Unprocessed blots)

Supplementary Fig. 1e

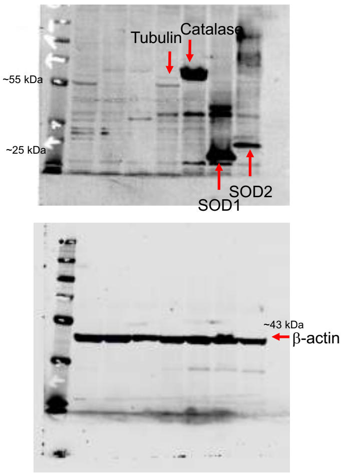

Supplementary Fig. 2a

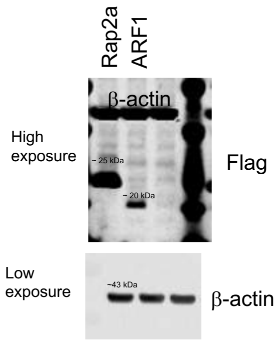

SUPPLEMENTARY FIGURE 7 (Unprocessed blots)

Figure 5i

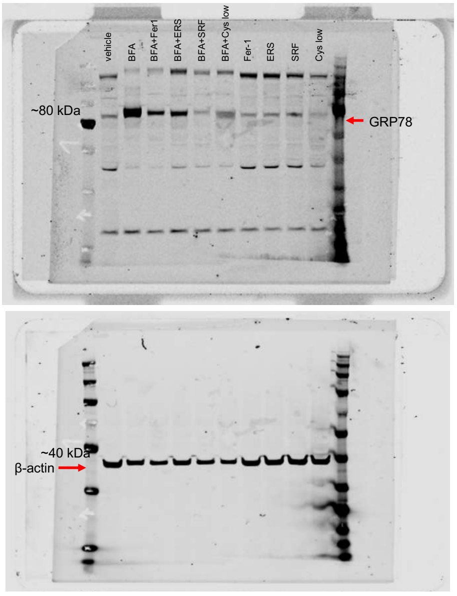

Supplementary Fig. 2d

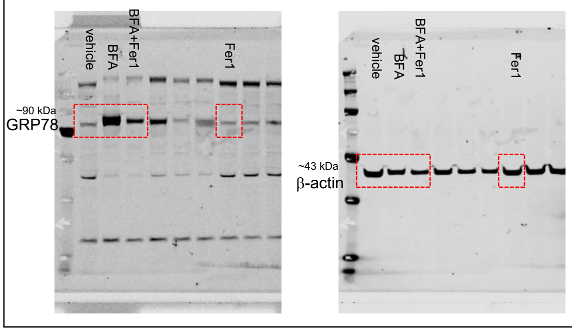

SUPPLEMENTARY FIGURE 7 (Unprocessed blots)

Supplementary Fig. 2f

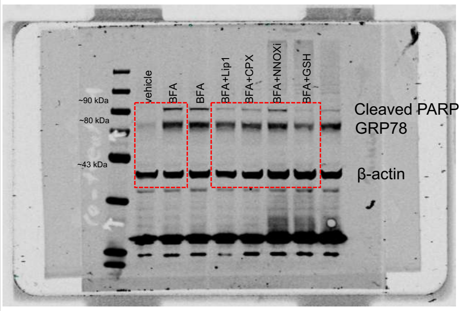

Supplementary Fig. 2i

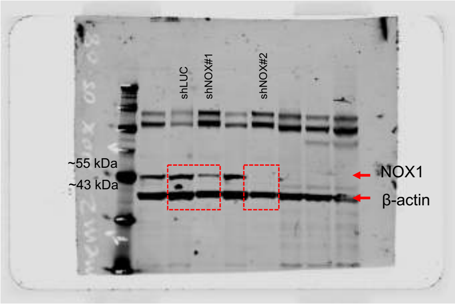

SUPPLEMENTARY FIGURE 7 (Unprocessed blots)

Supplementary Fig. 3a

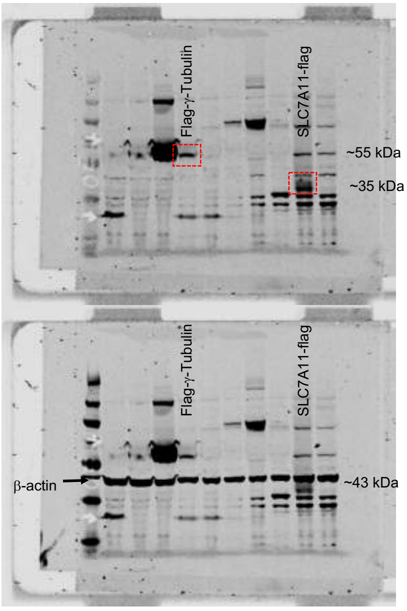

Supplementary Fig. 3b

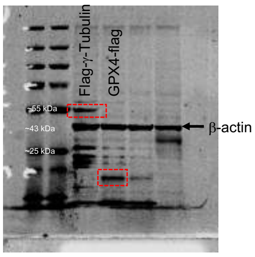

Supplementary Fig. 3d

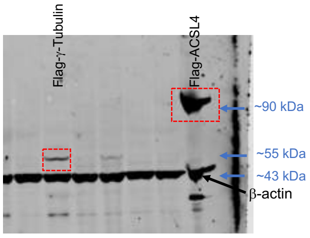

SUPPLEMENTARY FIGURE 7 (Unprocessed blots)

Supplementary Fig. 3e

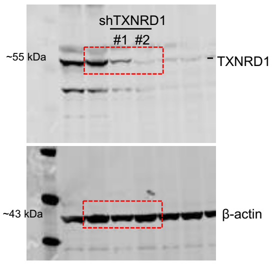

Supplementary Fig. 3g

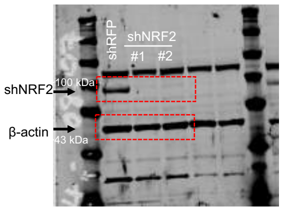

Supplementary Fig. 3h

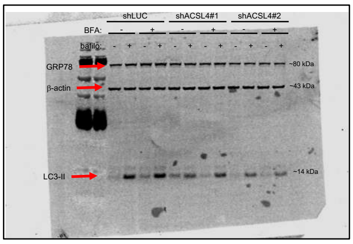

Supplementary Fig. 3i

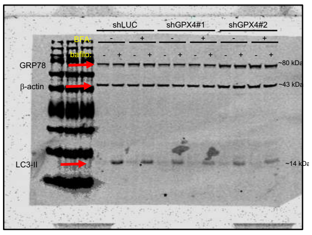

Supplementary Fig. 3j

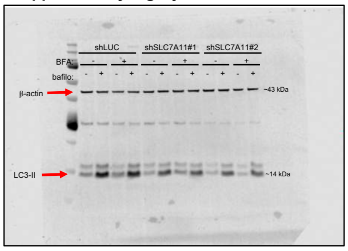

SUPPLEMENTARY FIGURE S7 (Unprocessed blots)

Supplementary Fig. 5g

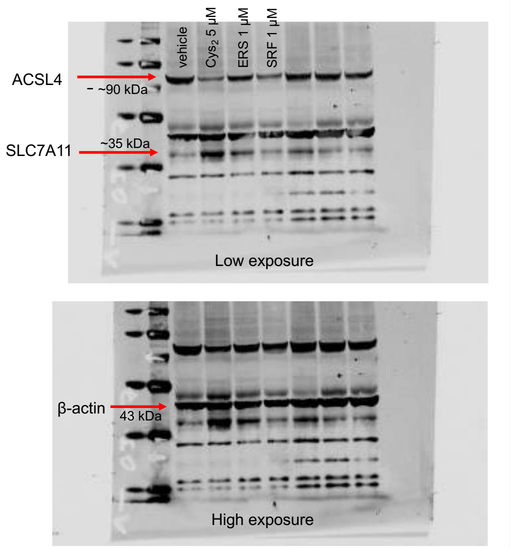

Supplement: Supplementary file 1 — Supplementary information [file 42003_2018_212_MOESM1_ESM.pdf]
